# Experiences of Discrimination and Everyday Racism Among Children and Adolescents With an Immigrant Background – Results of a Systematic Literature Review on the Impact of Discrimination on the Developmental Outcomes of Minors Worldwide

**DOI:** 10.3389/fpsyg.2022.805941

**Published:** 2022-05-09

**Authors:** Franka Metzner, Adekunle Adedeji, Michelle L.-Y. Wichmann, Zernila Zaheer, Lisa Schneider, Laura Schlachzig, Julia Richters, Susanne Heumann, Daniel Mays

**Affiliations:** ^1^Educational Science With a Focus on Special Education (“Emotional and Social Development”), University of Siegen, Siegen, Germany; ^2^Department of Medical Psychology, University Medical Center Hamburg-Eppendorf, Hamburg, Germany; ^3^Faculty of Humanities, North-West University, Mafikeng, South Africa; ^4^Faculty of Life Sciences, Hamburg University of Applied Sciences, Hamburg, Germany; ^5^Catholic University of Applied Sciences of North Rhine – Westphalia, Paderborn, Germany

**Keywords:** discrimination, children, adolescents, immigrant families, refugee families, development, systematic review

## Abstract

Experiences of discrimination such as everyday racism can negatively affect the mental and physical health of children and adolescents with an immigrant background and impair their integration process in the host societies. Although experiences of racism are part of the everyday life of many minors affected by the process of “Othering” (e.g., those with an immigrant background), an overview of empirical findings is missing for this age group worldwide. A systematic review was conducted to identify and analyze international research on the impact of discrimination on the developmental outcomes and integration of immigrant children and adolescents. Three scientific databases were systematically searched up to June 11, 2021. A total of *k* = 4,769 identified publications were reviewed based on inclusion and exclusion criteria in terms of the PICOS format by independent reviewers. Thirty-four primary studies published between 1998 and 2021 met all inclusion criteria. The samples examined were mainly migrant youth (*k* = 30), with only *k* = 2 studies assessing refugee youth and *k* = 1 study assessing both migrant and refugee youth. The majority of included studies assessed perceived discrimination, with only *k* = 1 study directly assessing experiences of racism. The association between discrimination or racism and developmental outcomes was assessed by the included studies within the three main topics of (a) mental and physical health-related outcomes (*k* = 30, e.g., self-esteem, depressive symptoms), (b) school-related outcomes (*k* = 6, e.g., academic achievement), and (c) other developmental outcomes (*k* = 13, e.g., misconduct or delinquency). Data collection procedures were implemented, and findings on minors’ developmental outcomes and integration process who experience discrimination and racism were summarized and discussed. The current review suggests experienced (racial) discrimination as a negative predictor of children and adolescents’ (mental) health-related outcomes, while no clear results could be found for the association between discrimination and school-related outcomes. A need for more empirical research focusing on the path and (possibly) indirect link between discrimination and children and adolescents’ school-related outcomes as well as resulting school recommendations and the chosen career path was derived.

**Systematic Review Registration:**

[https://www.crd.york.ac.uk/prospero/display_record.php?RecordID=260291], identifier [CRD42021260291].

## Introduction

Immigrant children and adolescents’ adaption and life outcomes have dominated recent research and political discourse on immigration (Human Rights Council, 2017). As the global migration trend continues to progress, many studies have outlined the perception and experience of discrimination as a socio-cultural factor crucial for child immigrants’ (first generation) as well as immigrants’ children’s (including the second generation) life outcomes (Canino and Spurlock, 2000; Vedder and van Geel, 2012; Brown, 2017).

While migration experiences often differ across various migrant groups, research has shown that immigrants are generally faced with common challenges that distinguish them from other minority groups (Bhugra and Becker, 2005; Wilkes and Wu, 2019). Migrants with a mostly non-transferable training or qualification, inadequate or no knowledge of the host language, limited or no work permit, a different understanding of social, economic and health resources or structures are faced with barriers in settlement and integration in the host community (Henderson, 2004; Grzymala-Kazlowska, 2018; Adedeji and Bullinger, 2019; Rogne et al., 2020). Furthermore, recent trends and patterns in migration have further reinforced a negative public perception of migrants as ‘desperate invaders’ or ‘poor victims of the ramping humanitarian crisis’ (De Haas, 2008; Schapendonk, 2012). This perception of migrants often collaborates with discrimination, exploitation and exclusion and may significantly impact the socioeconomic integration and the general well-being of migrants and their children.

Discrimination broadly refers to negative behaviors, actions or practices that exclude or merely differentiate between individuals or groups based on an ascribed or perceived trait (Al Ramiah et al., 2010). In recent explorations, researchers have continued to evaluate the effect of discrimination on health and how it impairs the integration process of minor immigrants. George and Bassani (2018), in a sample of immigrant children from China, Hong Kong, and the Philippines living in Canada, found that discrimination experienced by parents and family had a positive impact on the child’s health. They argued that, as newcomers, family cohesiveness and social support are enhanced and work to buffer children’s health when racial discrimination is perceived. Contrarily, Brown (2015) – in a report for the Migration Policy Institute in Washington DC – suggests that perceived ethnic discrimination by peers and teachers was, in fact, negatively related to children’s developmental outcomes. These developmental outcomes measure the successful adjustment of immigrant children and youth. This includes their mental and physical health, school-related outcomes, sense of social competence in peer relationships, general misconduct and delinquency, and a sense of mastery and control (de Leon Siantz, 1997; Coll and Magnuson, 2012; Dimitrova et al., 2016).

Regardless of the direction and findings, studies on discrimination agree that immigrant children experience discrimination through personal interactions or treatment and learning experiences at school. These experiences can be on a peer-level, e.g., in the form of peer exclusion (Brown and Chu, 2012), a teacher or guardian level, e.g., in the form of unjust treatment (Brown, 2015), and on a community level such as anti-immigrant sentiments and stereotypes (Rong and Brown, 2002). They may also experience structural discrimination through long-term institutional practices (e.g., school segregation, lack of engagement with parents). These experiences are projected to affect their personal development and academic trajectories in various capacities (Brown and Chu, 2012; Oxman-Martinez et al., 2012; Brown, 2015).

Immigrant children’s multidimensional exposure to discrimination reinforces the assumption that they are especially vulnerable to discrimination as they are exposed at all fronts (home, school, and social space). At the same time, they seek to explore their aptness into their new community, school, and culture (McCarthy, 1998; Chuang, 2011). This unique circumstance of immigrant children suggests different patterns of the impact of discrimination on life measures (de Leon Siantz, 1997; Portes et al., 2009). It further justifies the need for a comprehensive and systematic evaluation of discrimination as a social determinant of immigrant children and adolescents’ life outcomes.

However, several reviews on discrimination and life outcome in the last decade have focused on adult immigrants in specific populations, e.g., ethnic groups, including Asian Americans, African Americans, and Latino/a Americans (Panza et al., 2019; Kirkinis et al., 2021). These studies have centered on negative mental health, such as depression, as the primary health outcome (Sharac et al., 2010; Paradies et al., 2015). They summarized different pathways through which the experience and perception of discrimination have affected adult immigrants’ life outcomes, e.g., through socioeconomic disadvantages, adverse cognitive/emotional processes, social withdrawal, diminished participation in healthy behaviors, and physical injury due to racially motivated violence (Pascoe and Richman, 2009; Kirkinis et al., 2021).

It is evident that the experience and perception of discrimination plays a vital role in life outcome, integration and performance. This is more so for immigrant children exposed to multiple psychosocial stressors adapting to a new community, school, and social environment (Brown, 2015). Despite this, there have been very few attempts to provide an overview of empirical evidence on the effect of discrimination on immigrant children’s life outcomes and performance worldwide. Most of these reviews have focused broadly on minority groups. For example, Benner et al. (2018) investigated the relations between perceived discrimination by race/ethnicity or gender and well-being during adolescence. They analyzed 214 peer-reviewed publications and showed that greater perceptions of discrimination in youths were linked to more socioemotional distress, lower academic achievement, and more risky health behaviors (Benner et al., 2018).

Furthermore, Benner et al. (2018) found a stronger link between discrimination and socioemotional distress for adolescents of Asian descent than adolescents of African descent and a stronger connection between perceived discrimination and academics for adolescents of Latino descent vs. African descent. Similarly, other reviews have explored the association between perceived racial discrimination and mental health in different predefined settings (Bronstein and Montgomery, 2011; Borsch et al., 2019; Haft et al., 2021). However, these reviews generally ignored the unique experience and challenges faced by immigrant children and their potential effect on the associations between discrimination and developmental outcomes compared to children without immigrant backgrounds.

This study, therefore, aims to systematically explore existing empirical research to provide an overview of the association between experienced or perceived discrimination and life outcome and performances among immigrant children and adolescents worldwide. This necessary understanding is principal for developing interventions and research that promote and provide an essential support system to facilitate the adaptation of immigrant children (Human Rights Council, 2017). The objectives are to:

1) provide an overview of the scientific evidence on the association between discrimination and indicators of developmental outcomes among immigrant children and adolescents;
2) compare research findings of associating factors and indicators across several settings;
3) summarize the strengths and limitations of the current literature and discuss the methodological difficulties associated with measuring discrimination among immigrant children;
4) and identify future directions to advance this field of study.

## Materials and Methods

This systematic review synthesizes primary studies on the developmental consequences of discrimination and racism in immigrant children and adolescents worldwide. The current systematic review’s methodological approach is based on the guidelines for implementing and analyzing systematic reviews (Centre for Reviews and Dissemination, 2009; Moher et al., 2009; Higgins et al., 2019; Zawacki-Richter et al., 2020). These guidelines describe the research process and tools to summarize evidence relevant for decision-makers in evidence-based medicine and in the educational research field. This study also followed the Preferred Reporting Items for Systematic reviews and Meta-Analyses (PRISMA) 2020 statement (Page et al., 2020), including the recommended checklist for the publication of systematic reviews.

Our approach to this work was registered in the International prospective register of systematic reviews PROSPERO on July 7, 2021, after piloting the study selection process and before starting the formal screening of search results against eligibility criteria. Methods were captured in a review protocol created *a priori*, continuously updated during the research process, and uploaded to the PROSPERO website. Our registration and review protocol can be viewed on PROSPERO with the registration number CRD42021260291. The systematic review was conducted without funding.

### Inclusion and Exclusion Criteria

We selected our inclusion criteria (IC) and exclusion criteria (EC) in terms of the PICOS format (Higgins et al., 2019); see Table 1. We included studies examining children and adolescents (up to 21 years) being refugees, asylum seekers or immigrants in first (with own migration experiences) or second-generation (at least one parent with migration experience but without own migration experience) (IC 1). Studies examining members of ethnic minorities without first- or second-generation migration experience, participants older than 21 years or adopted children or adolescents with immigrant backgrounds were excluded (EC 1). The children and adolescents in the sample must have been examined concerning the independent variables discrimination or racism (IC 2). Discrimination involves exposure to a broad range of experiences, including those related to stigmatization, exclusion, social distancing, harassment, or violent acts within the individual or institutional contexts, committed by peers and non-peers. Racism or racial discrimination entails dominant group behaviors that result in minority ethnic groups being treated differently. Studies on bullying, which involves peer-to-peer maltreatment occurring in the school or as committed by peers from school but without indication for discrimination or racism, were excluded (EC 2). For inclusion of a study, at least one outcome related to development, well-being or health in children and adolescents (e.g., language or other academic skills, identity development, depression) had to have been investigated as a dependent variable (IC 3). Only original studies published as peer-reviewed journal articles with abstract, title and full-text in German or English were included (IC 4) with no date restrictions. We excluded unpublished studies, book chapters, congress contributions, and doctoral theses (EC 4.1). If the same sample was analyzed in two or more publications, we selected the publication with the more suitable objective or analysis and excluded the less relevant studies (EC 4.2). In addition, studies were excluded for which no full-texts were available (EC 4.3) or in which relevant results were not reported (EC 4.4). All study types that allow statements on the relationship between the independent and dependent variables were approved (IC 5). The PICOS criterion of the comparison group was not applicable for this study.

**TABLE 1:** Inclusion and exclusion criteria based on PICOS scheme.

**Patient/Population**	
IC 1	Children and adolescents (up to 21 years) being refugees, asylum seekers or immigrants in first (with own migration experiences) or second (at least one parent with migration experience but without own migration experience) generation.
EC 1	Members of ethnic minorities without first- or second-generation migration experience, participants older than 21 years, or adopted children or adolescents with an immigrant background
**Intervention**	
IC 2	Experiences of discrimination or racism. Discrimination involves exposure to a broad range of experiences, including those related to stigmatization, exclusion, social distancing, harassment, or violent acts within the individual or institutional contexts, committed by peers and non-peers. Racism or racial discrimination entails dominant group behaviors that result in subordinate ethnic groups being treated differently.
EC 2	Bullying involving peer-to-peer maltreatment occurring in the school or as committed by peers from school but without indication for discrimination or racism.
**Comparator**	
	*Not relevant (A comparator does not have to be present)*
**Outcome**	
IC 3	Outcomes related to the development, well-being or health in children and adolescents (e.g., language or other academic skills, identity development, and depression)
**Publication**	
IC 4	Original studies published as peer-reviewed journal articles with abstract, title and full-text in German or English language
EC 4.1	Unpublished studies, book chapters, congress contributions, doctoral theses
EC 4.2	The same sample analyzed in two or more publications
EC 4.3	Full-text not available
EC 4.4	Relevant results not presented in the full-text
**Study design**	
IC 5	All study types that allow statements on the relationship between the independent and dependent variable

### Information Sources

We searched for peer-reviewed primary literature in three scientific databases (Medline, Web of Science and PsycINFO) up to June 11, 2021. Additionally, we searched Google Scholar, reference lists of relevant publications, the German and the EU Clinical Trials Registers. We contacted leading researchers in the research field, asking for additional research, finishing the search process in August 2021.

### Search Strategy

Before starting the search process, we carried out preliminary searches. We tested the search string in the database Medline to optimize our methodology and focus. In addition, the PROSPERO database was searched to exclude possible overlap in content with studies that have not yet been published. This search in the PROSPERO database resulted in two ongoing systematic reviews with different thematic focus (CRD42018109787: Racial minority college students’ experiences of racial microaggressions and their well-being outcomes; registered 2018) or sample (CRD42020184055: Relationship between reported racism and health and well-being for children and adolescents; registered 2020). Our final search string (see Table 2) was searched in three databases. Additional search strategies were conducted to reduce publication bias.

**TABLE 2:** Terms used in systematic database literature search.

Category	English language
A: Discrimination	discriminat* OR racis* OR microagress*
B: Migration	refugee* OR asylum seek* OR evacuee* OR displaced OR immigrant* OR migrant* OR exile OR minority
C: Minors	child* OR young age OR infant OR adolesc* OR teen* OR famil* OR pediatr*
D: Developmental outcome	development* OR resilien* OR integration* OR language* OR academic* OR learn* OR health OR attachment OR vulnerab* OR social OR problem* OR difficult* OR cognitiv* OR self-regulat* OR well-being

### Screening and Study Selection

Citations identified from the systematic search were exported to the reference management tool EndNote 20. Duplicates were removed, and two independent reviewers (FM and AA) screened all titles and abstracts using the inclusion and exclusion criteria. Excluded references were labeled with the reason for exclusion. Articles that were labeled as “excluded” by both researchers were removed. Articles with conflicted votes (ineligible vs. potentially or probably eligible) were discussed until consensus was reached. The agreement rate was measured by determining the percentage of the sum of all matching “included” and “excluded” references, where the total number of all double screened references represented 100%. Interrater reliability was calculated using Cohen’s Kappa for about 10% of the studies (*k* = 487). The same two reviewers (FM and AA) screened the full-texts of all probably eligible articles using the same inclusion and exclusion criteria. Suppose consensus was not possible during the screening of title and abstract or full-text screening, a third or fourth reviewer (MW or JR), who had the casting vote, would be asked to independently screen the article.

### Data Extraction and Synthesis

The selection of characteristics to be extracted from the included primary studies was discussed with the research team, and a unanimous agreement was reached. After reviewing the full texts against the inclusion criteria, *k* = 34 studies were included in the systematic review. Using a structured table for data synthesis, two independent reviewers (AA and MW) extracted and coded relevant information for describing the studies included: the country where the study was conducted, methodology (e.g., study design, data collection, comparison group, assessed type of discrimination, assessed developmental outcomes), sample characteristics (e.g., size, age and gender distribution, country of origin or ethnicity, host country), and reported results for the association between discrimination or racism and developmental outcomes in the included studies. The independent reviewers extracted and coded information. In the case of mixed-methods studies, only quantitative methods and data were extracted. If the data extracted from the studies differed between the two reviewers, a third reviewer (FM or JR) was consulted. The two reviewers achieved an excellent agreement. No authors of studies included were contacted for further information or data.

### Assessment of Methodological Study Quality

Methodological quality assessment of included studies was completed using the Mixed Methods Appraisal Tool (MMAT; Hong et al., 2018). The tool was developed explicitly for quality assessment in systematic reviews that include primary research using quantitative, qualitative and mixed methods. Two independent reviewers (MW and FM) assessed methodological study quality, applying two screening questions and five quality criteria to each included study. Out of five possible study types assessable using the MMAT, only the categories “quantitative descriptive studies” and “quantitative non-randomized studies” were relevant to the current literature review. The screening questions and quality criteria were each rated as “met,” “not met,” or “not enough information available,” and methodological study quality was subsequently rated as “high,” “medium,” or “low.” The screening questions and applied quality criteria can be found in section “Methodological Quality Assessment of Included Studies.” For a “high” methodological quality rating, both screening questions and all quality criteria had to be met. A study was rated as having “medium” methodological quality if both screening questions and 3–4 quality criteria were met. A rating of “low” methodological quality was given if (a) one or more screening questions were not met or not enough information was available for a rating, or (b) both screening questions and 0–2 quality criteria were met. Conflicting assessments between reviewers were discussed until consensus was reached.

### Assessment of Risk of Bias

The primary studies were assessed with regard to their Risk of Bias (RoB) following the Grading of Recommendations Assessment, Development and Evaluation (GRADE) assessment (Zhang et al., 2019). RoB can be assessed for observational studies (Obs; non-randomized studies) and randomized-controlled trials (RCT). According to the GRADE assessment, some criteria increase the bias of study results. The GRADE manual was used to ensure the evaluation of RoB was transparent. The criteria were weighted, and a scale was created. In assessing the risk of bias for each RCT or observational study, the following limitations were considered: lack of allocation concealment, lack of blinding, incomplete coverage of patients and outcome events, selective outcome reporting, lack of development and application of appropriate eligibility criteria (inclusion of control population), incorrect measurement of both exposure and outcome, inadequate control of confounding factors, and incomplete or insufficiently short follow-up.

**FIGURE 1:**
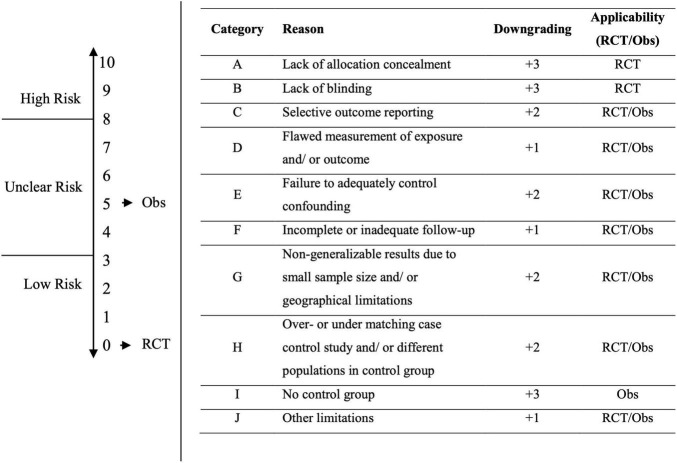
Risk of Bias assessment based on GRADE assessment. Starting point for RCTs is “Low Risk” with 0 points. Starting point for Obs is at “Unclear Risk” with 5 points. For each limitation, the corresponding downgrade (+1, +2, +3) is made on the scale (0 to 10), which results in the categorization of the risk of bias (Low, Unclear, High). Downgrading scores and categorization to low, unclear, and high risk were defined by the authors.

The risk of bias can be assessed along a scale (see Figure 1): low RoB (0 to 3 points), unclear RoB (4 to 7 points), and high RoB (8 to 10 points). The starting point for RCTs is “low risk,” with 0 points. The starting point for observational studies is “unclear risk,” with 5 points. For each limitation, the RoB increases by the corresponding degree (+1, +2, +3) on the scale (0 to 10), resulting in the categorization of RoB (low, unclear, high) (see Figure 1).

## Results

### Study Selection

**FIGURE 2:**
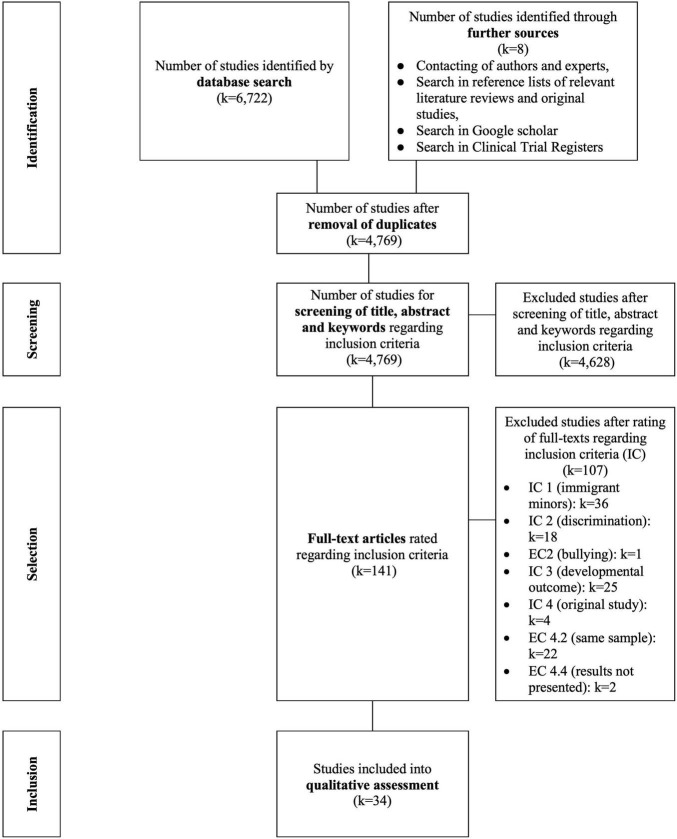
PRISMA-Flow-Diagram for the presentation of the study selection process according to Moher et al. (2009).

The study selection process for this systematic review is shown in Figure 2.

Three databases were searched, identifying *k* = 6,722 publications. Additional search strategies yielded further *k* = 8 references. After removing duplicates (*k* = 1,953), two independent researchers screened titles and abstracts of the remaining *k* = 4,769 studies (agreement rate: 93%, interrater reliability Cohen’s Kappa = 0.41). Records that did not meet the inclusion criteria were excluded (*k* = 4,628). The remaining *k* = 141 full-texts were screened by two independent researchers to assess eligibility (agreement rate: 62%, interrater reliability Cohen’s Kappa = 0.23). At the end of the study selection process, *k* = 34 records were included in the systematic review. The *k* = 107 excluded full-texts can be found in Supplementary Table 1.

### Study Characteristics of Included Studies

The included studies were published between 1998 and 2021 and originated in the United States (*k* = 11), Netherlands (*k* = 4), Canada and Portugal (*k* = 3 each), Norway (*k* = 2) and eleven other singular countries, nine of which were European (see Supplementary Table 2). Included studies mainly used cross-sectional designs (*k* = 27), with *k* = 6 studies using longitudinal and *k* = 1 study using both designs. A comparison sample was included in *k* = 7 studies, consisting of youth native to the host county (*k* = 6) or youth from the immigrants’ country of origin (*k* = 1). Funding was reported for *k* = 20 studies, with the most common sources being governments or government-funded research institutes (*k* = 10), foundations (*k* = 5) and educational institutions, e.g., universities (*k* = 5) (see Supplementary Table 2).

#### Description of Samples

Specific countries of origin were named in *k* = 23 studies, which included samples from 48 different countries located in Asia (*i* = 15), Africa and Europe (*i* = 11 each), North America (*i* = 5), Central America (*i* = 4) and South America (*i* = 2). Regions of origin and ethnic heritage (*k* = 5; e.g., “Latino,” “Black,” and “multi-ethnic”) were reported in *k* = 11 studies, including Africa, the American continents and Europe (*k* = 4 each), Asia and the Middle East (*k* = 2 each). The migrant and refugee samples were residing in 18 different host countries located in Europe (*i* = 13), North America (*i* = 2), East and West Asia (*i* = 2) as well as Australia (*i* = 1). Host countries were identical to the authors’ country in almost all studies (*k* = 30), with *k* = 2 studies each collecting data in a different country or in more than one country (see Supplementary Table 2).

The included samples were mainly migrant youth (*k* = 30), with only *k* = 2 studies assessing refugee youth and *k* = 1 study assessing both migrant and refugee youth (see Supplementary Table 2). Information regarding the proportion of first-generation immigrants and the duration of stay in the host countries was available in *k* = 26 and *k* = 11 studies. On average, these samples consisted of 45% first-generation immigrants (Range: 0–100%) and had resided in their host countries for 6.0 years (Range: 0–14 years). For the description of sample sizes and mean percentage of females and age, only data from the index group (i.e., migrant or refugee youth) and only Wave 2 data in longitudinal designs were considered. Sample sizes ranged from *n* = 95 to *n* = 4,288 (total: *N* = 22,115) with 52% females on average (Range: 33–67%; information missing in *k* = 9 studies). The migrant and refugee youth were 15.5 years old on average (Range: 10–21 years; information missing in *k* = 9 studies) (see Supplementary Table 2).

#### Assessed Types of Discrimination or Racism and Developmental Outcomes

The majority of included studies assessed perceived discrimination (*k* = 28), with only *k* = 1 study directly assessing experiences of racism. The proportion of migrant or refugee youth who had experienced at least one discriminatory or racist event was reported in only *k* = 9 studies (*M* = 36%, Range: 9–80%). Discrimination or racism was mainly assessed *via* self-report scales (*k* = 11), self-report (e.g., dichotomous items; *k* = 9) or by utilizing third-party-developed scales and questionnaires (*k* = 8). On average, 6.6 items were used to assess discrimination or racism (Range: 1–21 items) (see Supplementary Table 2). Concerning developmental outcomes, three main topics emerged: (a) mental and physical health-related, (b) school-related, and (c) other developmental outcomes. Because almost no studies included the proportion of migrant or refugee youth experiencing (negative) developmental outcomes in their results section, no percentages are reported below.

##### Mental and Physical Health-Related Developmental Outcomes

Almost all included studies (*k* = 31) assessed mental or physical health-related developmental outcomes, with depressive symptoms and self-esteem (*k* = 13 each) as well as externalizing problems (*k* = 5) being the most frequent. Depressive symptoms were mostly assessed using the Center for Epidemiological Studies Depression Scale (CES-D; *k* = 7, incl. adaptations) or self-report scales (*k* = 3). The instruments had an average of 14.7 items (Range: 5–27 items). Self-esteem was mainly measured using Rosenberg’s Self-Esteem Scale (RSES; *k* = 10), with instruments having an average of 8.1 items (Range: 3–10 items). Externalizing problems were mainly assessed using the Youth Self-Report (YSR; *k* = 2) or self-report scales (*k* = 2); *M* = 8.5 items (Range: 1–17) (see Supplementary Table 2).

##### School-Related Developmental Outcomes

School-related outcomes were assessed in *k* = 6 studies, which measured academic achievement or performance. Other school-related outcomes were academic self-concept and positive school value (*k* = 1 each). Academic achievement or performance was measured using the youths’ grades or self-report scales (*k* = 4 each). The self-report scales had an average of 2.8 items (Range: 2–4 items) (see Supplementary Table 2).

##### Other Developmental Outcomes

Overall *k* = 15 studies assessed a range of other developmental outcomes, with social relationships (*k* = 7) and misconduct or delinquent behavior (*k* = 5) being the most frequent. Social relationships were mainly measured with self-report scales (*k* = 5), with an average of 5.5 items across all utilized instruments (Range: 1–10 items). Misconduct or delinquent behavior was measured using self-report scales (*k* = 2) or other instruments, including the SDQ or YSR (*k* = 1 each). An average of 11.8 items was used to assess misconduct or delinquent behavior (Range: 5–18 items) (see Supplementary Table 2).

### Methodological Quality Assessment of Included Studies

All of the *k* = 34 studies included in this systematic literature review passed methodological quality screening. The methodological quality was subsequently rated high for *k* = 2 studies, medium for *k* = 24 studies and low for *k* = 8 studies (see Table 3). The high number of medium and low ratings mainly resulted from included studies not reporting enough information on their sample (e.g., sampling process, inclusion and exclusion criteria, non-responders) to make a clear judgment.

**TABLE 3:** Adapted rating of the methodological quality of included studies (*k* = 34) based on the Mixed Methods Appraisal Tool (MMAT; Hong et al., 2018).

	Screening		Quantitative descriptive studies						Quantitative non-randomized studies				
	Clear RQ^1^	Data address RQ^2^	Sampling^3^	Representativity^4^	Measurements^5^	Non-response bias^6^	Statistical analysis^7^	Representativity^8^	Measurements^9^	Outcome data^10^	Confounders^11^	Exposure^12^	Overall quality assessment
Adriaanse et al., 2014	+	+						+	+	+	+	+	HIGH
Astell-Burt et al., 2012	+	+						0	+	0	+	+	MEDIUM
Balkaya et al., 2019	+	+	+	0	+	−	+						MEDIUM
Becerra et al., 2015	+	+	+	0	+	0	+						MEDIUM
Behnke et al., 2011	+	+	0	−	+	−	+						LOW
Beiser and Hou, 2016	+	+	+	+	+	0	+						MEDIUM
Borges et al., 2011	+	+	+	0	0	+	+						MEDIUM
Cano et al., 2015	+	+	+	0	+	0	+						MEDIUM
Chun and Chung, 2011	+	+						0	+	−	+	+	MEDIUM
Correa-Velez et al., 2010	+	+	+	0	+	0	+						MEDIUM
Cristini et al., 2011	+	+	+	0	+	0	+						MEDIUM
D’hondt et al., 2016	+	+	+	+	+	+	+						HIGH
El Bouhaddani et al., 2019	+	+						0	+	0	+	+	MEDIUM
Ellis et al., 2008	+	+	+	0	+	+	+						MEDIUM
Espinosa, 2020	+	+	0	0	+	0	+						LOW
Guerra et al., 2019	+	+						−	+	0	+	+	MEDIUM
Kauff et al., 2017	+	+	+	0	+	0	+						MEDIUM
Kiang et al., 2015	+	+	+	0	+	0	+						MEDIUM
Kogan et al., 2019	+	+	+	0	+	0	+						MEDIUM
Liebkind and Jasinskaja-Lahti, 2000	+	+	+	0	+	0	+						MEDIUM
Neto and Barros, 2000	+	+						0	+	0	+	+	MEDIUM
Neto, 2001	+	+	0	0	+	0	+						LOW
Oczlon et al., 2021	+	+	0	0	+	0	+						LOW
Okamoto et al., 2009	+	+	+	+	+	0	+						MEDIUM
Oppedal et al., 2004	+	+	+	0	+	0	+						MEDIUM
Oppedal, 2011	+	+	+	+	+	−	+						MEDIUM
Oxman-Martinez et al., 2012	+	+	0	0	+	0	+						LOW
Özdemir and Stattin, 2013	+	+	+	0	+	+	+						MEDIUM
Pantzer et al., 2006	+	+						0	+	0	+	+	MEDIUM
Potochnick and Perreira, 2010	+	+	+	+	+	−	+						MEDIUM
Sabatier and Berry, 2008	+	+	0	0	+	0	+						LOW
Tummala-Narra and Claudius, 2013	+	+	+	0	+	0	+						MEDIUM
Verkuyten, 1998	+	+	0	0	+	0	+						LOW
Verkuyten and Thijs, 2004	+	+	0	0	+	0	+						LOW

*^1^Are there clear research questions (RQ)?, ^2^Do the collected data allow to address the research questions (RQ)?, ^3^Is the sampling strategy relevant to address the research question?, ^4^Is the sample representative of the target population?, ^5^Are the measurements appropriate?, ^6^Is the risk of non-response bias low?, ^7^Is the statistical analysis appropriate to answer the research question?, ^8^Are the participants representative of the target population?, ^9^Are measurements appropriate regarding both the outcome and exposure?, ^10^Are there complete outcome data?, ^11^Are the confounders accounted for in the design and analysis?, ^12^During the study period, is the intervention administered (or exposure occurred) as intended? + quality criterion met, − quality criterion not met, 0 = not enough information available. Overall assessment of methodological study quality: HIGH, both screening questions and all quality criteria met; MEDIUM, both screening questions met and 1–2 quality criteria not met/not enough information available; LOW, at least 1 screening question not met or more than 2 quality criteria not met/not enough information available.*

### Risk of Bias Assessment of Included Studies

Results of the RoB assessment show that *k* = 21 studies had a high RoB, while *k* = 13 studies showed an unclear RoB. No studies had a low RoB (see Table 4). This result can be attributed to the fact that no studies used a randomized controlled design and that most observational studies did not include a control group.

**TABLE 4:** Risk of Bias assessment for included studies based on GRADE assessment (*k* = 34).

	Risk of Bias	Design	Reasons for downgrading
Adriaanse et al., 2014	UNCLEAR (6)	Obs	Population was under matched (J)
Astell-Burt et al., 2012	UNCLEAR (5)	Obs	Inadequate follow-up (F)
Balkaya et al., 2019	HIGH (8)	Obs	No control group (I)
Becerra et al., 2015	HIGH (8)	Obs	Flawed measurement (D) Failure to adequately control confounding (E)
Behnke et al., 2011	HIGH (10)	Obs	Failure to adequately control confounding (E) No control group (I)
Beiser and Hou, 2016	UNCLEAR (6)	Obs	Small amounts of variance in mental health scores (J)
Borges et al., 2011	HIGH (8)	Obs	No control group (I)
Cano et al., 2015	HIGH (8)	Obs	No control group (I)
Christini, 2011	HIGH (9)	Obs	Failure to adequately control confounding (E) Non-generalizable due to small sample size (G)
Chun and Chung, 2011	HIGH (9)	Obs	No control group (I) Flawed measurement (D)
Correa-Velez et al., 2010	HIGH (10)	Obs	No control group (I) Failure to adequately control confounding (E)
D’hondt et al., 2016	HIGH (8)	Obs	No control group (I)
El Bouhaddani et al., 2019	HIGH (10)	Obs	Flawed measurement (D) Non-generalizable due to small sample size (G) No control group (I)
Ellis et al., 2008	UNCLEAR (7)	Obs	Non-generalizable due geographical limitations (G)
Espinosa, 2020	HIGH (8)	Obs	Flawed measurement (D) Failure to adequately control confounding (E)
Guerra et al., 2019	HIGH (9)	Obs	Cross-sectional design (J) No control group (I)
Kauff et al., 2017	UNCLEAR (6)	Obs	Flawed measurement (D)
Kiang et al., 2015	UNCLEAR (7)	Obs	Non-generalizable due geographical limitations (G)
Kogan et al., 2019	UNCLEAR (6)	Obs	Cross-sectional design (J)
Liebkind et al., 2004	UNCLEAR (5)	Obs	/
Neto and Barros, 2000	UNCLEAR (5)	Obs	/
Neto, 2001	HIGH (9)	Obs	Flawed measurement (D) No control group (I)
Oczlon et al., 2021	UNCLEAR (7)	Obs	Cross-sectional design (J) Flawed measurement (D)
Okamoto et al., 2009	HIGH (9)	Obs	Failure to adequately control confounding (E) Non-generalizable due geographical limitations (G)
Oppedal et al., 2004	HIGH (8)	Obs	No control group (I)
Oppedal, 2011	HIGH (9)	Obs	No control group (I) Flawed measurement (D)
Oxman-Martinez et al., 2012	HIGH (8)	Obs	Cross-sectional design (J) Non-generalizable due geographical limitations (G)
Özdemir and Stattin, 2013	HIGH (9)	Obs	Non-generalizable due to small sample size for each group (G) Failure to adequately control confounding (E)
Pantzer et al., 2006	HIGH (10)	Obs	Non-generalizable due geographical limitations (G) No control group (I) Flawed measurement (D)
Potochnick and Perreira, 2010	UNCLEAR (5)	Obs	/
Sabatier and Berry, 2008	UNCLEAR (5)	Obs	/
Tummala-Narra and Claudius, 2013	HIGH (10)	Obs	Non-generalizable due to small sample size (G) Flawed measurement (D) No control group (I)
Verkuyten and Thijs, 2004	HIGH (9)	Obs	Flawed measurement (D) No control group (I)
Verkuyten and Thijs, 2006	UNCLEAR (5)	Obs	/

*Obs, observational study design; RCT, randomized controlled trial.*

### Reported Results for the Association Between Discrimination or Racism and Developmental Outcomes

The included studies assessed the association between discrimination or racism and developmental outcomes within the three main topics of (a) mental and physical health-related outcomes (*k* = 30), (b) school-related outcomes (*k* = 6), and (c) other developmental outcomes, e.g., interpersonal relationships or misconduct and delinquency (*k* = 13). To assess the association between discrimination or racism and developmental outcomes, the included studies most frequently used correlations (*k* = 17), linear regressions (*k* = 16) and logistic regressions (*k* = 7). Reported results and methods for data analysis are presented in Supplementary Table 3.

#### Findings on Mental and Physical Health-Related Outcomes

Mental and physical health-related outcomes include self-esteem, depressive symptoms, externalizing problems, internalizing problems, global (mental) health, well-being and other mental and physical health-related outcomes. In the following, reported results for these categories are summarized.

##### Self-Esteem

The association between discrimination or racism and self-esteem was assessed in *k* = 12 studies, with *k* = 11 studies reporting at least one statistically significant association. Correlation coefficients ranged between *r* = −0.03 and *r* = −0.39. Reported standardized coefficients ranged between β = −0.03 and β = −0.25; information was missing for *j* = 6 examined associations (see Supplementary Table 3). Overall, these results indicate a weak to moderate negative association between discrimination or racism and migrant or refugee youths’ self-esteem.

##### Depressive Symptoms

Overall *k* = 10 studies assessed the association between discrimination or racism and depressive symptoms. Significant results were reported in *k* = 9 studies for at least one examined association. Correlation coefficients ranged between *r* = 0.08 and *r* = 0.45. Reported standardized coefficients ranged between β = 0.10 and β = 0.40. Non-standardized regression coefficients ranged between *B* = 0.02 and *B* = 1.88, with standard errors between SE = 0.02 and SE = 0.59 (see Supplementary Table 3). Overall, the reported results indicate a moderately high positive association between discrimination or racism and depressive symptoms.

##### Externalizing Problems

A total of *k* = 5 studies examined the relationship between discrimination or racism and externalizing problems among immigrant children and adolescents. Significant associations were reported in *k* = 4 studies for at least one examined association. The correlation coefficients suggest a weak (*r* = 0.02) to strong (*r* = 0.56) association between both variables. Similarly, results from regression analyses returned beta scores ranging from β = 0.14 to β = 0.31, suggesting discrimination or racism as a significant strong predictor of immigrant children and adolescents’ externalizing problems (see Supplementary Table 3).

##### Internalizing Problems

The association between discrimination or racism and internalizing problems was examined in *k* = 4 studies. Significant associations were reported in *k* = 3 studies for at least one examined association. The results suggest a positive weak to strong association with correlation coefficients ranging from *r* = 0.09 to *r* = 0.51 and regression coefficients ranging from β = 0.13 to β = 0.54 (see Supplementary Table 3).

##### Global (Mental) Health and Well-Being

The relationship between global (mental) health and discrimination or racism was assessed in *k* = 4 studies. Significant results were reported in *k* = 3 studies for at least one assessed association. The results suggested a moderate inverse association between global (mental) health and discrimination or racism. Similarly, *k* = 3 studies assessed the association between discrimination or racism and immigrant children and adolescents’ well-being. Results from the three studies suggested weak to moderate associations (see Supplementary Table 3).

##### Other Mental or Physical Health-Related Outcomes

Overall, *k* = 10 studies examined the association between discrimination or racism and other mental or physical health-related outcomes. Significant associations with discrimination were reported in *k* = 6 studies for psychotic and delusional experiences, acculturative or adaptive stress, posttraumatic stress disorder (PTSD), overall psychological symptoms, health-related quality of life as well as global self-worth (see Supplementary Table 3).

#### Findings on School-Related Outcomes

The impact of discrimination or racism on school-related outcomes was examined in *k* = 6 studies focusing on academic achievement and performance (see Supplementary Table 3). Of the six studies, *k* = 4 studies focused on school achievement. Two of these studies reported a significant association between at least one measure of school-related outcomes and discrimination. Results from these studies return correlation coefficients ranging from *r* = 0.00 and *r* = −0.94 and standardized coefficients from regression analysis ranging from β = −0.003 and β = −0.01. Similarly, *k* = 1 study examined the association between discrimination and academic self-concept, while *k* = 1 study examined the association between discrimination and positive school value. Both studies reported moderately significant inverse associations for immigrant children and adolescents (see Supplementary Table 3).

#### Findings on Other Developmental Outcomes

Several studies (*k* = 13) examined the association between discrimination or racism and other developmental outcomes. From the thirteen studies, *k* = 4 explored the association between children and adolescents’ relationships with others and discrimination or racism. From these four studies, *k* = 3 reported at least one significant association. Similarly, four studies explored discrimination as a predictor of misconduct and delinquency, of which *k* = 3 studies reported at least 1 significant association. Another three (*k* = 3) examined the association between sense of mastery and control and discrimination. Two of these studies reported a significant association with correlation coefficients ranging from *r* = −0.01 to *r* = −0.28. Studies assessing substance use (*k* = 2), life satisfaction (*k* = 2), and sense of competence (*k* = 2) reported significant associations with discrimination (see Supplementary Table 3).

## Discussion

### Summary of Key Findings

In summary, the current review suggests experienced (racial) discrimination as a negative predictor of children and adolescents’ mental and physical health-related outcomes. The experience of discrimination is associated with lower self-esteem, lower well-being and poorer overall (mental) health. Similarly, the experience of discrimination predicts depressive symptoms as well as externalizing and internalizing problems. The focus of the majority of studies included in this review seems to be on self-esteem and depression; other outcomes were examined in only a few studies (5 maximum). This finding is particularly interesting because it suggests that (racial) discrimination is regarded as an individual problem first. On the contrary, however, structural racism and discrimination affect society.

Findings on the association between discrimination and school-related outcomes show no clear results. While most studies did not find significant associations between discrimination and academic performance, one study assessing school value and academic self-concept reported discrimination as a significant predictor of children and adolescent school performance. These conflicting results project the complexity of discrimination as a determinant of school outcomes and potential indirect effects through other social and economic characteristics. (Racial) Discrimination can be multi-faceted and may be hard to recognize or hidden. Hence, a significant conclusion of this review is to emphasize the need for more empirical research focusing not only on the path and (possibly) indirect link between discrimination and children and adolescents’ school-related outcomes but also on resulting school recommendations and children and adolescents’ chosen career paths.

At least in the German school context, these findings are unexpected: The German Program for International Student Assessment (PISA) study (Gomolla and Radtke, 2009) found that children with migrant backgrounds perform significantly worse at school than children without migrant background, with potential reasons mainly relating to the subjects of study and their family characteristics. However, explanations that see the German school system itself as a decisive reason for migrants’ poorer school performance are on the rise: Institutional discrimination is assumed, which, for example, makes it difficult for migrants to receive recommendations for a higher qualified school career (Gomolla and Radtke, 2009). A possible explanation for why the association between discrimination and academic performance remained inconclusive in this systematic review could be that most of the studies were conducted outside of Germany and that institutional discrimination in schools does not have such a high profile outside of the German context. At the same time, the study conducted by Astell-Burt et al. (2012) draws attention to the fact that study participants also experience racism in school.

Further findings link perceived or experienced discrimination to other developmental outcomes, e.g., a low ability to conduct for interpersonal relationships, misconduct or delinquency, a lower sense of mastery and control, higher substance use, as well as lower life satisfaction and sense of competence. Since only very few studies assessed each of these outcomes, more research is necessary to draw clear conclusions about the association with discrimination and racism. Ultimately, the question of whether poor school performance or the ability to conduct interpersonal relationships and delinquent behavior also contains the danger of rewriting a racist (and classist) story. Especially school performances or delinquent behavior can be highly constructed and prioritized, generating inequalities. Thus, the studies included in this review and their data bodies need to be contextualized in constituting power and inequality relations.

### Methodological Limitations, Strengths and Suggestions for Future Research

Some methodological limitations have to be considered when interpreting the results of this systematic review. First, only English and German language studies were included. Therefore, it is possible that other relevant publications in other languages were missed. Second, despite measures to reduce publication bias (e.g., through multiple and independent screening processes), the possibility that the included articles reported only significant results could not be ruled out. Third, most of the articles included in this review had cross-sectional study designs. Results, therefore, primarily reflect experiences and developmental outcomes at a specific time and with a very subjective view.

Furthermore, the cross-sectional study designs show significant correlations but do not allow for causal statements between experiences of discrimination and developmental outcomes. More longitudinal studies and parallel-group trials would be necessary to comprehensively assess whether discrimination affects developmental outcomes. However, discrimination is predominantly a personal experience and cannot be randomly allotted to persons, thereby complicating the possibility of RCTs with this specific sample and research question. However, more longitudinal studies may be of interest to assess the long-term effects of discrimination and how immigrant children and adolescents’ experiences change over time. Quasi-experimental studies, such as non-randomized controlled studies, before-and-after studies, or interrupted time series that compare children and adolescents with and without an immigrant background could yield interesting findings with greater explanatory power about causal relationships.

In addition, qualitative and representative quantitative studies on the experiences of discrimination among immigrant children and adolescents, considering different settings and forms – due to various, possibly intertwined characteristics and the perspective of caregivers (e.g., parents, educators or teachers) – seem necessary.

Fourth, the methodological quality assessment resulted in overall medium quality ratings, mainly due to the included studies not reporting enough information. The assessment tool used (MMAT; Hong et al., 2018) was developed primarily for the quality assessment of clinical or medical studies. As mentioned above, the included studies were mainly cross-sectional designs and therefore examined populations within a non-clinical trial. Nonetheless, we chose to use the MMAT despite this limitation due to the potential to assess the methodological quality of different study types within one review. Fifth, the current review was limited to experiences of discrimination at the individual level. Thus, it did not capture institutional and structural forms of discrimination. Although personal experiences of discrimination may be significant for specific outcomes, exploring institutional and structural discrimination might provide an even more prominent understanding of discrimination as a predictor of life outcomes. Furthermore, more primary research capturing the interactions of different forms of discrimination, i.e., intersectionality (Crenshaw, 1989), would be necessary to grasp the complexity of discrimination and its effects in various areas of life. Findings on (intersectional) discrimination experiences of immigrated minors can contribute to a critical view of discrimination in psychotherapeutic and pedagogical work. On the other hand, it emphasizes the psychological or physical consequences of the stress and potential trauma associated with discrimination experiences in childhood and adolescence (Schlachzig et al., 2021).

Considering that the primary research subjects here are children and adolescents, exploring their experience of intergenerational discrimination might provide more refined information that capitalizes on their young age and their supposedly inferior position in the generational order. While the current systematic review focuses on first- and second-generation immigrant children, it did not rule out discrimination as a significant factor for developmental outcomes in other third or more-generation immigrant children and adolescents. In a similar review, Benner et al. (2018) confirm discrimination as a predictor of life outcomes among minority groups. However, primary research would be needed to fully understand how the experience of discrimination affects first- and second-generation immigrant children differently from other minority groups. Despite the extensive search, only very few studies on the relationship between experiences of discrimination and developmental outcomes among very young and refugee children could be identified in the present systematic review. Future studies should address these research gaps, presumably due to the ethical and methodological challenges of collecting scientific data from these subgroups. This will provide in-depth insights into these particularly vulnerable children with special protection needs.

Results from the current review project various associations that allows implications to be drawn (e.g., connection of well-being, school assessment and the experience of discrimination). Diverse samples from multiple countries of origin and host countries were included. This provides a more complete foundation for new directions in understanding discrimination and developmental outcome. It is worth noting that the studies included in this review are particularly informative and use a range of perspectives and standardized instruments crucial for the empirical exploration of discrimination and developmental outcome.

## Conclusion

This systematic review illustrates the relationship between discrimination and developmental outcomes among immigrant children and adolescents. It found that the experience of discrimination mainly affects (mental) health and social relationships. The review highlights the consequences of discrimination and confirms that the different manifestations of discrimination, such as modernized racism and microaggression, must be considered alongside deeper explorations of how they affect developmental outcomes. The findings confirm that schools are not free of discrimination and point to critical practical implications for parents, teachers, policymakers and schools. It is crucial to address the question of which structures protect and prevent the experience of discrimination and promote self-esteem, well-being, and overall (mental) health. Schlachzig et al. (2021) emphasize the importance for teachers and social workers to reflect on one’s own racialized patterns of interpretation and action. They suggest; examining one’s own speech practices, listening and acknowledging talks about experiences of discrimination, and countering and not reproducing discrimination as practices that protect affected children. In addition, the current findings support that for training or treatment practice among immigrant children and adolescents, teachers, social workers, and other stakeholders should be oriented toward a discrimination-critical approach. This will facilitate perceiving and reducing individual and structural disadvantages in therapeutic care and different life outcomes. More adaptable diversity and anti-discrimination concepts are urgently needed in (pre-)schools to protect children and adolescents from discrimination. These concepts should include and institutionalize preventive, recognizing and intervening measures (e.g., Federal Anti-Discrimination Agency, 2019).

## Data Availability Statement

The original contributions presented in the study are included in the article/Supplementary Material, further inquiries can be directed to the corresponding author/s.

## Author Contributions

FM contributed to conceptualization, supervision study and writing process, piloting, study selection, review registration, writing methods, creating Figure 2 and Tables 1, 2, assessing study quality, third review data extraction, and writing discussion. AA contributed to conceptualization, piloting, study selection, supervision of the review process, writing the introduction and discussion, and keeping a study protocol. MW contributed to writing results, creating Tables 3, 4 and Supplementary Tables 1–3, qualitative assessment/data extraction, assessing and describing study quality. ZZ contributed to supporting piloting and study selection, documentation of the study process, writing the study protocol. LiS and LaS contributed to conceptualization, writing discussion, reviewing the manuscript. JR contributed to supporting qualitative assessment/data extraction, third review for study selection, supporting of creating Tables 3, 4 and Supplementary Tables 1–3, formatting the manuscript. SH contributed to writing methods, assessing and describing risk of bias, reviewing the manuscript, creating Figure 1. DM contributed to conceptualization, reviewing the manuscript. All authors contributed to the article and approved the submitted version.

## Conflict of Interest

The authors declare that the research was conducted in the absence of any commercial or financial relationships that could be construed as a potential conflict of interest.

## Publisher’s Note

All claims expressed in this article are solely those of the authors and do not necessarily represent those of their affiliated organizations, or those of the publisher, the editors and the reviewers. Any product that may be evaluated in this article, or claim that may be made by its manufacturer, is not guaranteed or endorsed by the publisher.
