# Supplementary material for: Experiences of Discrimination and Everyday Racism Among Children and Adolescents With an Immigrant Background – Results of a Systematic Literature Review on the Impact of Discrimination on the Developmental Outcomes of Minors Worldwide
Source: Front Psychol. 2022 May 9;13:805941. doi: 10.3389/fpsyg.2022.805941 (PMC9126147; doi:10.3389/fpsyg.2022.805941)
Supplement: Supplementary file 1 [file Data_Sheet_1.docx]

Table 1: List of excluded research after full-text screening

|  | Reference | Reason for exclusion | Respective PICOS criteria |
| --- | --- | --- | --- |
| 1 | Adair, J. K., Colegrove, K. S. S., & McManus, M. E. (2017). How the Word Gap Argument Negatively Impacts Young Children of Latinx Immigrants’ Conceptualisations of Learning. *Harvard Educational Review*, 87(3), 309-334. doi:10.17763/1943-5045-87.3.309 | Original studies published as peer-reviewed journal articles with abstract, title and full-text in German or English language | IC4 |
| 2 | Altinyelken, H. K. (2009). Migration and self-esteem: A qualitative study among internal migrant girls in Turkey. *Adolescence*, 44(173),  149-163. | Outcomes related to the development, well-being or health in children and adolescents | IC3 |
| 3 | Altinyelken, H. K. (2009). Educational Challenges of Internal Migrant Girls: a case study among primary school children in Turkey. *Research in Comparative and International Education,* 4(2), 211-228. doi:10.2304/rcie.2009.4.2.211 | Outcomes related to the development, well-being or health in children and adolescents | IC3 |
| 4 | Anderson, A. T., Luartz, L., Heard-Garris, N., Widaman, K., & Chung, P. J. (2020). The Detrimental Influence of Racial Discrimination on Child Health in the United States. *Journal of the National Medical Association*, 112(4), 411-422. doi:10.1016/j.jnma.2020.04.012 | Children and adolescents (up to 21 yrs) being refugees, asylum seekers or immigrants in the first or second generation | IC1 |
| 5 | Andrighetto, L., Durante, F., Lugani, F., Volpato, C., & Mirisola, A. (2013). Obstacles to intergroup contact: when outgroup partner's anxiety meets  perceived ethnic discrimination. *The British journal of social psychology*, 52(4), 781-792. doi:10.1111/bjso.12039 | Outcomes related to the development, well-being or health in children and adolescents | IC3 |
| 6 | Appel, M. (2012). Anti-Immigrant Propaganda by Radical Right Parties and the Intellectual Performance of Adolescents. *Political Psychology*, 33(4), 483-493. doi:10.1111/j.1467-9221.2012.00902.x | Outcomes related to the development, well-being or health in children and adolescents | IC3 |
| 7 | Arora, P. G., & Wheeler, L. A. (2018). Depressive symptoms in Mexican-origin adolescents: Interrelations between school and family contexts. *Contemporary School Psychology*, 22(3), 266-278. doi:10.1007/s40688-017-0150-1 | The same sample analysed in two or more publications | EC4.2 |
| 8 | Assari, S., Akhlaghipour, G., Boyce, S., Bazargan, M., & Caldwell, C. H. (2020). Parental Human Capital and Adolescents' Executive Function: Immigrants' Diminished Returns. *Medical research archives*, 8(10). doi:10.18103/mra.v8i10.2235 | Experiences of discrimination or racism | IC2 |
| 9 | Aurelius, G. (1979). Adjustment and behaviour of Finnish and Southern European immigrant children in Stockholm. I. The teachers' assessment. *Scandinavian journal of social medicine*, 7(3), 105-113. doi:10.1177/140349487900700302 | Children and adolescents (up to 21 yrs) being refugees, asylum seekers or immigrants in the first or second generation | IC1 |
| 10 | Ayon, C., Marsiglia, F. F., & Bermudez-Parsai, M. (2010). Latino Family Mental Health: Exploring the Role of Discrimination and Familismo. *Journal of Community Psychology*, 38(6), 742-756. doi:10.1002/jcop.20392 | Children and adolescents (up to 21 yrs) being refugees, asylum seekers or immigrants in the first or second generation | IC1 |
| 11 | Ayon, C., & Philbin, S. P. (2017). "Tu No Eres de Aqui": Latino Children's Experiences of Institutional and Interpersonal Discrimination and  Microaggressions. *Social Work Research*, 41(1), 19-30. doi:10.1093/swr/svw028 | Children and adolescents (up to 21 yrs) being refugees, asylum seekers or immigrants in the first or second generation | IC1 |
| 12 | Bacallao, M. L., & Smokowski, P. R. (2013). Obstacles to Getting Ahead: How Assimilation Mechanisms Impact, Undocumented Mexican Immigrant Families.*Social Work in Public Health*, 28(1), 1-20. doi:10.1080/19371910903269687 | Experiences of discrimination or racism | IC2 |
| 13 | Baker, J. (2013). Just Kids? Peer Racism in a Predominantly White City. *Refuge*, 29(1), 75-85. | Outcomes related to the development, well-being or health in children and adolescents | IC3 |
| 14 | Basanez, T., Unger, J. B., Soto, D., Crano, W., & Baezconde-Garbanati, L. (2013). Perceived discrimination as a risk factor for depressive symptoms and substance use among Hispanic adolescents in Los Angeles. *Ethnicity & Health,* 18(3), 244-261. doi:10.1080/13557858.2012.713093 | Children and adolescents (up to 21 yrs) being refugees, asylum seekers or immigrants in the first or second generation | IC1 |
| 15 | Baysu, G., Celeste, L., Brown, R., Verschueren, K., & Phalet, K. (2016). Minority adolescents in ethnically diverse schools: Perceptions of equal treatment buffer threat effects. *Child Development,* 87(5), 1352-1366. doi:10.1111/cdev.12609 | The same sample analysed in two or more publications | EC4.2 |
| 16 | Becares, L., Nazroo, J., & Kelly, Y. (2015). A longitudinal examination of maternal, family, and area-level experiences of racism on children's socioemotional development: Patterns and possible explanations. *Social Science & Medicine*, 142, 128-135. doi:10.1016/j.socscimed.2015.08.025 | Children and adolescents (up to 21 yrs) being refugees, asylum seekers or immigrants in the first or second generation | IC1 |
| 17 | Beiser, M., Taa, B., Fenta-Wube, H., Baheretibeb, Y., Pain, C., & Araya, M. (2012). A comparison of levels and predictors of emotional problems among  preadolescent Ethiopians in Addis Ababa, Ethiopia, and Toronto, Canada. *Transcultural Psychiatry*, 49(5), 651-677. doi:10.1177/1363461512457155 | The same sample analysed in two or more publications | IC4.2 |
| 18 | Bennett, M., Roche, K. M., Huebner, D. M., & Lambert, S. F. (2020). School Discrimination and Changes in Latinx Adolescents' Internalizing and  Externalising Symptoms. *Journal of Youth and Adolescence*, 49(10), 2020-2033. doi:10.1007/s10964-020-01256-4 | Children and adolescents (up to 21 yrs) being refugees, asylum seekers or immigrants in the first or second generation | IC1 |
| 19 | Berry, J. W. & Sabatier, C. (2010). Acculturation, discrimination, and adaption among second generation immigrant youth in Montreal and Paris. *International Journal of Intercultural Relations*, 34, 191-207. doi:10.1016/j.ijintrel.2009.11.007 | The same sample analysed in two or more publications | EC4.2 |
| 20 | Birman, D., Trickett, E., & Buchanan, R. M. (2005). A Tale of Two Cities: Replication of a Study on the Acculturation and Adaptation of Immigrant Adolescents From the Former Soviet Union in a Different Community Context. *American Journal of Community Psychology,* 35(1-2), 83-101. doi:10.1007/s10464-005-1891-y | Outcomes related to the development, well-being or health in children and adolescents | IC3 |
| 21 | Boutry-Avezou, V., Sabatier, C., & Brisset, C. (2007). Psychological well-being at school, social adaptation and perceived discrimination in second generation immigrant adolescents. Bien-etre, adaptation sociale et discrimination a l'ecole. Perception des adolescents issus de l'immigration., 7(3), 205-216. | Original studies published as peer-reviewed journal articles with abstract, title and full-text in German or English language | IC4 |
| 22 | Brabant, L. H., Lapierre, S., Damant, D., Dube-Quenum, M., Lessard, G., & Fournier, C. (2016). Immigrant Children: Their Experience of Violence at School and Community in Host Country. *Children & Society*, 30(3), 241-251. doi:10.1111/chso.12131 | Experiences of discrimination or racism | IC2 |
| 23 | Cardoso, J. B., Brabeck, K., Capps, R., Chen, T., Giraldo-Santiago, N., Huertas, A., & Mayorga, N. A. (2021). Immigration Enforcement Fear and Anxiety in Latinx High School Students: The Indirect Effect of Perceived Discrimination. *Journal of Adolescent Health*, 68(5), 961-968. doi:10.1016/j.jadohealth.2020.08.019 | Children and adolescents (up to 21 yrs) being refugees, asylum seekers or immigrants in the first or second generation | IC1 |
| 24 | Cervantes, R. C., Fisher, D. G., Cordova, D., & Napper, L. E. (2012). The Hispanic Stress Inventory--Adolescent Version: a culturally informed psychosocial assessment. *Psychological assessment*, 24(1), 187-196. doi:10.1037/a0025280 | Experiences of discrimination or racism | IC2 |
| 25 | Correa-Velez, I., Gifford, S. M., & McMichael, C. (2015). The persistence of predictors of well-being among refugee youth eight years after resettlement  in Melbourne, Australia. *Social Science & Medicine*, 142, 163-168. doi:10.1016/j.socscimed.2015.08.017 | Experiences of discrimination or racism | IC2 |
| 26 | Dangmann, C., Solberg, O., & Andersen, P. N. Health-related quality of life in refugee youth and the mediating role of mental distress and post-migration stressors. *Quality of Life Research*. doi:10.1007/s11136-021-02811-7 | Experiences of discrimination or racism | IC2 |
| 27 | Davis, A. N., Carlo, G., Schwartz, S. J., Unger, J. B., Zamboanga, B. L., Lorenzo-Blanco, E. I., . . . Soto, D. (2016). The Longitudinal Associations Between Discrimination, Depressive Symptoms, and Prosocial Behaviors in US Latino/a Recent Immigrant Adolescents. *Journal of Youth and Adolescence*, 45(3), 457-470. doi:10.1007/s10964-015-0394-x | The same sample analyzed in two or more publications | EC4.2 |
| 28 | Davis, A. N., McGinley, M., Carlo, G., Schwartz, S. J., Unger, J. B., Des Rosiers, S. E., . . . Soto, D. Examining discrimination and familism values as longitudinal predictors of prosocial behaviours among recent immigrant adolescents. *International Journal of Behavioral Development.* doi:10.1177/01650254211005561 | Outcomes related to the development, well-being or health in children and adolescents | IC3 |
| 29 | D'Hondt, F., Eccles, J. S., Houtte, M., & Stevens, P. A. J. (2016). Perceived ethnic discrimination by teachers and ethnic minority students' academic futility: Can parents prepare their youth for better or worse?. *Journal of Youth and Adolescence*, 45(6), 1075-1089. doi:10.1007/s10964-016-0428-z | Children and adolescents (up to 21 yrs) being refugees, asylum seekers or immigrants in the first or second generation | IC1 |
| 30 | Ellis, B. H., MacDonald, H. Z., Klunk-Gillis, J., Lincoln, A., Strunin, L., & Cabral, H. J. (2010). Discrimination and Mental Health Among Somali Refugee Adolescents: The Role of Acculturation and Gender. *American Journal of Orthopsychiatry,* 80(4), 564-575. doi:10.1111/j.1939-0025.2010.01061.x | The same sample analyzed in two or more publications | EC4.2 |
| 31 | Fan, X.-h., & Chen, F.-j. (2012). Perceived discrimination and depression: Moderating coping and social support in migrant children. *Chinese Journal of Clinical Psychology*, 20(4), 539-542. | Children and adolescents (up to 21 yrs) being refugees, asylum seekers or immigrants in the first or second generation | IC1 |
| 32 | Firat, M., & Noels, K. A. Perceived discrimination and psychological distress among immigrants to Canada: The mediating role of bicultural identity  orientations. *Group Processes & Intergroup Relations.* doi:10.1177/1368430221990082 | Children and adolescents (up to 21 yrs) being refugees, asylum seekers or immigrants in the first or second generation | IC1 |
| 33 | George, M. A., Bassani, C., & Armstrong, R. W. (2012). Influence of Perceived Racial Discrimination on Health and Behaviour of Immigrant Children in British Columbia. *International Journal of Population Research*. doi:10.1155/2012/274963 | The same sample analyzed in two or more publications | EC4.2 |
| 34 | George, M. A., & Bassani, C. (2018). Influence of Perceived Racial Discrimination on the Health of Immigrant Children in Canada. *Journal of international migration and integration*, 19(3), 527-540. doi:10.1007/s12134-018-0539-3 | The same sample analyzed in two or more publications | EC4.2 |
| 35 | Goldberg, D., & Hodes, M. (1992). The poison of racism and the self-poisoning of adolescents. *Journal of Family Therapy*, 14(1), 51-67. doi:10.1046/j..1992.00442.x | Children and adolescents (up to 21 yrs) being refugees, asylum seekers or immigrants in the first or second generation | IC1 |
| 36 | Grigsby, T. J., Forster, M., Meca, A., Zamboanga, B. L., Schwartz, S. J., & Unger, J. B. (2018). Cultural stressors, identity development, and substance use attitudes among Hispanic immigrant adolescents. *Journal of Community Psychology*, 46(1), 117-132. doi:10.1002/jcop.21920 | The same sample analyzed in two or more publications | EC4.2 |
| 37 | Han, Y. (2014). Differential Size of the Discrimination-Depression Relationship Among Adolescents of Foreign-Born Parents in the US.  *Child & Youth Care Forum*, 43(6), 763-781. doi:10.1007/s10566-014-9265-y | Same sample analyzed in two or more publications | EC4.2 |
| 38 | Hao, Z., & Cui, L. (2015). The role of intergroup permeability on Chinese migrant children's social integration. *Social Behavior and Personality: An International Journal,* 43(2), 303-314. doi:10.2224/sbp.2015.43.2.303 | Children and adolescents (up to 21 yrs) being refugees, asylum seekers or immigrants in the first or second generation | IC1 |
| 39 | Isseri, S., Muthukrishna, N., & Philpott, S. C. (2018). Immigrant children's geographies of schooling experiences in South Africa. *Educational Research for Social Change,* 7(2), 39-56.  doi:10.17159/2221-4070/2018/v7i2a3 | Original studies published as peer-reviewed journal articles with abstract, title and full-text in German or English language | IC4 |
| 40 | Jasinskaja-Lahti, I., & Liebkind, K. (2001). Perceived discrimination and psychological adjustment among Russian-speaking immigrant adolescents in Finland. *International Journal of Psychology,* 36(3), 174-185. doi:10.1080/00207590042000074 | The same sample analyzed in two or more publications | EC4.2 |
| 41 | Jasinskaja-Lahti, I., Liebkind, K., Horenczyk, G., & Schmitz, P. (2003). The interactive nature of acculturation: Perceived discrimination, acculturation attitudes and stress among young ethnic repatriates in Finland, Israel and Germany. *International Journal of Intercultural Relations,* 27(1), 79-97. doi:10.1016/S0147-1767%2802%2900061-5 | Outcomes related to the development, well-being or health in children and adolescents | IC3 |
| 42 | Jiang, S., & Dong, L. (2020). The effects of teacher discrimination on depression among migrant adolescents: Mediated by school engagement and  moderated by poverty status. *Journal of Affective Disorders*, 275, 260-267. doi:10.1016/j.jad.2020.07.029 | Children and adolescents (up to 21 yrs) being refugees, asylum seekers or immigrants in the first or second generation | IC1 |
| 43 | Jore, T., Oppedal, B., & Biele, G. (2020). Social anxiety among unaccompanied minor refugees in Norway. The association with pre-migration trauma and post-migration acculturation related factors. *Journal of Psychosomatic Research*, 136. doi:10.1016/j.jpsychores.2020.110175 | Children and adolescents (up to 21 yrs) being refugees, asylum seekers or immigrants in the first or second generation | IC1 |
| 44 | Jugert, P., Pink, S., Fleischmann, F., & Leszczensky, L. (2020). Changes in Turkish- and Resettler-origin Adolescents' Acculturation Profiles of Identification: A Three-year Longitudinal Study from Germany. *Journal of Youth and Adolescence*, 49(12), 2476-2494. doi:10.1007/s10964-020-01250-w | Children and adolescents (up to 21 yrs) being refugees, asylum seekers or immigrants in the first or second generation | IC1 |
| 45 | Khanlou, N., Koh, J. G., & Mill, C. (2008). Cultural identity and experiences of prejudice and discrimination of Afghan and Iranian immigrant youth. *International Journal of Mental Health and Addiction,* 6(4), 494-513. doi:10.1007/s11469-008-9151-7 | Outcomes related to the development, well-being or health in children and adolescents | IC3 |
| 46 | Kiang, L., Supple, A. J., Stein, G. L., & Gonzalez, L. M. (2012). Gendered Academic Adjustment among Asian American Adolescents in an Emerging Immigrant Community. *Journal of Youth and Adolescence,* 41(3), 283-294. doi:10.1007/s10964-011-9697-8 | The same sample analyzed in two or more publications | EC4.2 |
| 47 | Kiang, L., Espino-Perez, K., & Stein, G. L. (2020). Discrimination, Skin Color Satisfaction, and Adjustment among Latinx American Youth. *Journal of Youth and Adolescence*, 49(10), 2047-2059. doi:10.1007/s10964-020-01244-8 | Outcomes related to the development, well-being or health in children and adolescents | IC3 |
| 48 | Kienzler, H., Wenzel, T., & Shaini, M. (2019). Vulnerability and psychosocial health  experienced by repatriated children in Kosovo.  *Transcultural Psychiatry*, 56(1), 267-286. doi:10.1177/1363461518802992 | Experiences of discrimination or racism | IC2 |
| 49 | Kim, J., Suh, W., Kim, S., & Gopalan, H. (2012). Coping strategies to manage acculturative stress: Meaningful activity participation, social support, and positive emotion among Korean immigrant adolescents in the USA. *International Journal of Qualitative Studies on Health and Well-being*, 7. doi:10.3402/qhw.v7i0.18870 | Experiences of discrimination or racism | IC2 |
| 50 | Kim, J., Suh, W., & Heo, J. (2014). Do Korean Immigrant Adolescents Experience Stress-Related Growth During Stressful Intergroup Contact and  Acculturation? *Journal of Humanistic Psychology*, 54(1), 3-27. doi:10.1177/0022167812468614 | Outcomes related to the development, well-being or health in children and adolescents | IC3 |
| 51 | Kim, J.-H., Kim, J. Y., & Kim, S.-S. (2016). School Violence, Depressive Symptoms, and Help-seeking Behavior: A Gender-stratified Analysis of Biethnic Adolescents in South Korea. *Journal of*  *preventive medicine and public health,* 49(1), 61-68. doi:10.3961/jpmph.15.060 | Experiences of discrimination or racism | IC2 |
| 52 | Kira, I. A., Lewandowski, L., Ashby, J. S., Somers, C., Chiodo, L., & Odenat, L. (2014). Does bullying victimization suppress IQ? The effects of bullying  victimization on IQ in Iraqi and African American adolescents: A traumatology perspective. *Journal of Aggression, Maltreatment & Trauma,* 23(5), 431-453. doi:10.1080/10926771.2014.904463 | Bullying | EC2 |
| 53 | Kira, I. A., Lewandowski, L., Chiodo, L., & Ibrahim, A. (2014). Advances in systemic trauma theory: Traumatogenic dynamics and consequences of backlash as a multi-systemic trauma on Iraqi refugee Muslim adolescents. *Psychology*, 5(5), 389-412. doi:10.4236/psych.2014.55050 | Experiences of discrimination or racism | IC2 |
| 54 | Kiramba, L. K., Onyewuenyi, A. C., Kumi-Yeboah, A., & Sallar, A. M. (2020). Navigating multiple worlds of Ghanaian-born immigrant adolescent girls in US urban schools. International Journal of Intercultural Relations, 77, 46-57. doi:10.1016/j.ijintrel.2020.04.003 | Outcomes related to the development, well-being or health in children and adolescents | IC3 |
| 55 | Kokkonen, P., Athanasopoulou, C., Leino-Kilpi, H., & Sakellari, E. (2021). Secondary School Pupils' Mental Wellbeing Is Associated with Belonging to  a Perceived Minority and Experiencing Discrimination. *Children-Basel,* 8(2). doi:10.3390/children8020071 | Children and adolescents (up to 21 yrs) being refugees, asylum seekers or immigrants in the first or second generation | IC1 |
| 56 | Kubiliene, N., Yan, M. C., Kumsa, M. K., & Burman, K. (2015). The response of youth to racial discrimination: implications for resilience theory. *Journal of Youth Studies*, 18(3), 338-356.  doi:10.1080/13676261.2014.963535 | Children and adolescents (up to 21 yrs) being refugees, asylum seekers or immigrants in the first or second generation | IC1 |
| 57 | Kunyu, D. K., Schachner, M. K., Juang, L. P., Schwarzenthal, M., & Aral, T. (2021). Acculturation hassles and adjustment of adolescents of immigrant descent: Testing mediation with a self-determination theory approach. *New directions for child and adolescent development*. doi:10.1002/cad.20408 | Children and adolescents (up to 21 yrs) being refugees, asylum seekers or immigrants in the first or second generation | IC1 |
| 58 | Kwan, M. Y., Gordon, K. H., & Minnich, A. M. (2018). An examination of the relationships between acculturative stress, perceived discrimination, and eating disorder symptoms among ethnic minority college students. *Eating Behaviors,* 28, 25-31. doi:10.1016/j.eatbeh.2017.12.003 | Children and adolescents (up to 21 yrs) being refugees, asylum seekers or immigrants in the first or second generation | IC1 |
| 59 | Lam, A. M. C., Tsoi, K. W., & Chan, T. S. (2005). Adolescent Chinese immigrants in Hong Kong: A comparison with locally born students and factors associated with their psychological well-being. *International Journal of Adolescent Medicine and Health*, 17(2), 157-168. doi:10.1515/IJAMH.2005.17.2.157 | Children and adolescents (up to 21 yrs) being refugees, asylum seekers or immigrants in the first or second generation | IC1 |
| 60 | Lee, M., Kim, Y., & Madyun, N. i. (2018). Do relational and structural characteristics of negative school environments independently predict immigrant adolescents' academic achievement? *Social Psychology of Education: An International*  *Journal*, 21(3), 539-563. doi:10.1007/s11218-018-9427-0 | Same sample analyzed in two or more publications | EC4.2 |
| 61 | Li, S. D., & Xia, Y. (2018). Understanding the link between discrimination and juvenile delinquency among Chinese migrant children. *Journal of Contemporary Criminal Justice*, 34(2), 128-147. doi:10.1177/1043986218761959 | Children and adolescents (up to 21 yrs) being refugees, asylum seekers or immigrants in the first or second generation | IC1 |
| 62 | Liebkind, K., Jasinskaja-Lahti, I., & Solheim, E. (2004). Cultural identity, perceived discrimination, and parental support as determinants of immigrants' school adjustments: Vietnamese youth in Finland. *Journal of Adolescent Research*, 19(6), 635-656. doi:10.1177/0743558404269279 | The same sample analyzed in two or more publications | EC4.2 |
| 63 | Liu, X., Zhao, J., & Shen, J. (2013). Perceived discrimination and subjective wellbeing  among urban migrant children: The effect of mediator and moderator. *Acta Psychologica Sinica*, 45(5), 568-584. doi:10.3724/SP.J.1041.2013.00568 | Original studies published as peer-reviewed journal articles with abstract, title and full-text in German or English language | IC4 |
| 64 | Liu, D. Y., Yu, X. B., Wang, Y. C., Zhang, H. Q., & Ren, G. F. (2014). The impact of perception of discrimination and sense of belonging on the loneliness of the children of Chinese migrant workers: a structural equation modelling analysis. *International Journal of Mental Health Systems*, 8.  doi:10.1186/1752-4458-8-52 | Children and adolescents (up to 21 yrs) being refugees, asylum seekers or immigrants in the first or second generation | IC1 |
| 65 | Liu, X., & Zhao, J. X. (2016). Chinese Migrant Adolescents' Perceived Discrimination and Psychological Well-Being: The Moderating Roles of Group Identity and the Type of School. *Plos One,* 11(1). doi:10.1371/journal.pone.0146559 | Children and adolescents (up to 21 yrs) being refugees, asylum seekers or immigrants in the first or second generation | IC1 |
| 66 | Lo, C. C., Hopson, L. M., Simpson, G. M., & Cheng, T. C. (2017). Racial/Ethnic Differences in Emotional Health: A Longitudinal Study of Immigrants' Adolescent Children. *Community Mental Health Journal*, 53(1), 92-101. doi:10.1007/s10597-016-0049-8 | The same sample analyzed in two or more publications | EC4.2 |
| 67 | Lopez, A., & Shen, C. (2021). Predictors of Self-Esteem Among Mexican Immigrant Adolescents: An Examination of Well-Being Through a Biopsychosocial Perspective. *Child and Adolescent Social Work Journal*, 38(3), 347-358. doi:10.1007/s10560-020-00682-4 | The same sample analyzed in two or more publications | EC4.2 |
| 68 | Lopez, W. D., LeBron, A. M. W., Graham, L. F., & Grogan-Kaylor, A. (2016). Discrimination and Depressive Symptoms Among Latina/o Adolescents of Immigrant Parents. *International Quarterly of Community Health Education*, 36(2), 131-140. doi:10.1177/0272684x16628723 | The same sample analyzed in two or more publications | EC4.2 |
| 69 | Maes, M., Stevens, G., & Verkuyten, M. (2014). Perceived Ethnic Discrimination and Problem Behaviors in Muslim Immigrant Early Adolescents: Moderating Effects of Ethnic, Religious, and National Group Identification. *Journal of Early Adolescence,* 34(7), 940-966. doi:10.1177/0272431613514629 | Outcomes related to the development, well-being or health in children and adolescents | IC3 |
| 70 | Marks, A. K., McKenna, J. L., & Garcia Coll, C. (2018). National immigration receiving contexts: A critical aspect of native-born, immigrant, and refugee youth well-being. *Special Issue: Youth and Migration: What Promotes and What Challenges Their Integration?*, 23(1), 6-20. doi:10.1027/1016-9040/a000311 | Experiences of discrimination or racism | IC2 |
| 71 | Medvedeva, M. (2010). Perceived Discrimination and Linguistic Adaptation of Adolescent Children of Immigrants. *Journal of Youth and Adolescence*, 39(8), 940-952. doi:10.1007/s10964-009-9434-8 | The same sample analyzed in two or more publications | EC4.2 |
| 72 | Miconi, D., Altoe, G., Salcuni, S., Di Riso, D., Schiff, S., & Moscardino, U. (2018). Discrimination and Externalizing Problems Among Moroccan- and Romanian-Origin Early Adolescents in Italy: Moderating Role of Cultural Orientations and Impulse Control. *Cultural Diversity & Ethnic Minority Psychology,* 24(3), 374-388. doi:10.1037/cdp0000192 | Outcomes related to the development, well-being or health in children and adolescents | IC3 |
| 73 | Montgomery, E. (2008). Long-term effects of organized violence on young Middle Eastern refugees' mental health. *Social Science & Medicine*, 67(10), 1596-1603. doi:10.1016/j.socscimed.2008.07.020 | Experiences of discrimination or racism | IC2 |
| 74 | Montgomery, E., & Foldspang, A. (2008). Discrimination, mental problems and social adaptation in young refugees. *European Journal of Public Health*, 18(2), 156-161. doi:10.1093/eurpub/ckm073 | Outcomes related to the development, well-being or health in children and adolescents | IC3 |
| 75 | Motti-Stefanidi, F., Pavlopoulos, V., Obradovic, J., Dalla, M., Takis, N., Papathanassiou, A., & Masten, A. S. (2008). Immigration as a risk factor for adolescent adaptation in Greek urban schools. *European Journal of Developmental Psychology*, 5(2), 235-261. doi: 10.1080/17405620701556417 | Experiences of discrimination or racism | IC2 |
| 76 | Muller, L. R. F., Gossmann, K., Schmid, R. F., Rosner, R., & Unterhitzenberger, J. (2021). A pilot study on ecological momentary assessment in asylum-seeking children and adolescents resettled to Germany: Investigating compliance, post-migration factors, and the relation between daily mood, sleep patterns, and mental health. *Plos One*, 16(2). doi:10.1371/journal.pone.0246069 | Outcomes related to the development, well-being or health in children and adolescents | IC3 |
| 77 | Neto, F. (2009). Predictors of Mental Health Among Adolescents From Immigrant Families in Portugal. *Journal of Family Psychology,* 23(3), 375-385. doi:10.1037/a0015831 | Children and adolescents (up to 21 yrs) being refugees, asylum seekers or immigrants in the first or second generation | IC1 |
| 78 | Neto, J., & Neto, F. (2014). Acculturation and behaviour problems of adolescents from African immigrant families in Portugal. Acculturation:  Psychology, processes and global perspectives., 167-180. | Outcomes related to the development, well-being or health in children and adolescents | IC3 |
| 79 | Nho, C. R., Yoon, S., Seo, J., & Cui, L. (2019). The mediating effect of perceived social support between depression and school adjustment in refugee children in South Korea. Children and Youth Services Review, 106. doi:10.1016/j.childyouth.2019.104474 | Experiences of discrimination or racism | IC2 |
| 80 | Niwa, E. Y., Way, N., & Hughes, D. L. (2014). Trajectories of Ethnic-Racial Discrimination Among Ethnically Diverse Early Adolescents: Associations With Psychological and Social Adjustment. *Child Development*, 85(6), 2339-2354. doi:10.1111/cdev.12310 | Children and adolescents (up to 21 yrs) being refugees, asylum seekers or immigrants in the first or second generation | IC1 |
| 81 | Okoye, H. U., & Saewyc, E. (2021). Fifteen-year trends in self-reported racism and link with health and well-being of African Canadian adolescents: a  secondary data analysis. *International Journal for Equity in Health,* 20(1). doi:10.1186/s12939-021-01446-x | Children and adolescents (up to 21 yrs) being refugees, asylum seekers or immigrants in the first or second generation | IC1 |
| 82 | Oppedal, B., Roysamb, E., & Heyerdahl, S. (2005). Ethnic group, acculturation, and psychiatric problems in young immigrants. *Journal of Child Psychology and Psychiatry*, 46(6), 646-660. doi:10.1111/j.1469-7610.2004.00381.x | Experiences of discrimination or racism | IC2 |
| 83 | Oxman-Martinez, J., & Choi, Y. R. (2014). Newcomer Children: Experiences of Inclusion and Exclusion, and Their Outcomes*. Social*  *Inclusion*, 2(4), 23-37. doi:10.17645/si.v2i4.133 | Experiences of discrimination or racism | IC2 |
| 84 | Pahl, K., & Way, N. (2006). Longitudinal trajectories of ethnic identity among urban Black and Latino adolescents. *Child Development*, 77(5), 1403-1415. doi:10.1111/j.1467-8624.2006.00943.x | Outcomes related to the development, well-being or health in children and adolescents | IC3 |
| 85 | Pavez-Soto, I., Ortiz-Lopez, J. E., Sepulveda, N., Jara, P., & Olguin, C. (2019). Racialization of Haitian Migrant Childhood in Chilean Schools. *Interciencia*, 44(7), 414-420. | Outcomes related to the development, well-being or health in children and adolescents | IC3 |
| 86 | Perreira, K. M., Fuligni, A., & Potochnick, S. (2010). Fitting In: The Roles of Social Acceptance and Discrimination in Shaping the Academic Motivations of Latino Youth in the US Southeast. *Journal of Social Issues*, 66(1), 131-153. doi:10.1111/j.1540-4560.2009.01637.x | Children and adolescents (up to 21 yrs) being refugees, asylum seekers or immigrants in the first or second generation | IC1 |
| 87 | Perreira, K. M., & Ornelas, I. (2013). Painful Passages: Traumatic Experiences and Post-Traumatic Stress among US Immigrant Latino Adolescents and their Primary Caregivers. *International Migration Review*, 47(4), 976-1005. doi:10.1111/imre.12050 | The same sample analyzed in two or more publications | EC4.2 |
| 88 | Petersen, L.-E., Dunnbier, U., & Morgenroth, O. (2012). Ethnic identity and ethnicity-related stress in accompanied and unaccompanied adolescent immigrants: Does the family work as social capital for adolescent immigrants? *Psychology,* 3(4), 370-377. doi:10.4236/psych.2012.34052 | Experiences of discrimination or racism | IC2 |
| 89 | Pieloch, K. A., Marks, A. K., & Coll, C. G. (2018). A person-centred exploration of children of immigrants' social experiences and their school-based wellbeing. *Applied Developmental Science*, 22(2), 110-124. doi:10.1080/10888691.2016.1225500 | Relevant results not presented in the full-text | EC4.4 |
| 90 | Potochnick, S., Perreira, K. M., & Fuligni, A. (2012). Fitting In: The Roles of Social Acceptance and Discrimination in Shaping the Daily Psychological Well-Being of Latino Youth. *Social Science Quarterly,* 93(1), 173-190. doi:10.1111/j.1540-6237.2011.00830.x | Children and adolescents (up to 21 yrs) being refugees, asylum seekers or immigrants in the first or second generation | IC1 |
| 91 | Qu, D., Chen, C., Kouros, C. D., & Yu, N. X. (2021). Congruence and discrepancy in migrant children's and mothers' perceived discrimination: Using response surface analysis to examine the effects on psychological distress. *Applied Psychology: Health and Well-Being*, No-Specified. doi:http://dx.doi.org/10.1111/aphw.12249 | Children and adolescents (up to 21 yrs) being refugees, asylum seekers or immigrants in the first or second generation | IC1 |
| 92 | Ringel, S., Ronell, N., & Getahune, S. (2005). Factors in the integration process of adolescent immigrants - The case of Ethiopian Jews in Israel. *International Social Work*, 48(1), 63-+.  doi:10.1177/0020872805048709 | Outcomes related to the development, well-being or health in children and adolescents | IC3 |
| 93 | Rios-Salas, V., & Larson, A. (2015). Perceived discrimination, socioeconomic status, and mental health among Latino adolescents in US immigrant  families. *Children and Youth Services Review*, 56, 116-125. doi:10.1016/j.childyouth.2015.07.011 | Relevant results not presented in the full-text | EC4.4 |
| 94 | Roche, C., & Kuperminc, G. P. (2012). Acculturative stress and school belonging among Latino youth. *Hispanic Journal of Behavioral Sciences*, 34(1), 61-76. doi:10.1177/0739986311430084 | Outcomes related to the development, well-being or health in children and adolescents | IC3 |
| 95 | Romero, A. J., & Roberts, R. E. (2003). Stress within a bicultural context for adolescents of Mexican descent. *Cultural Diversity and Ethnic Minority Psychology*, 9(2), 171-184. doi:10.1037/1099-9809.9.2.171 | Experiences of discrimination or racism | IC2 |
| 96 | Schachner, M. K., Van de Vijver, F. J. R., & Noack, P. (2018). Acculturation and School Adjustment of Early-Adolescent Immigrant Boys and Girls in  Germany: Conditions in School, Family, and Ethnic Groups. *Journal of Early Adolescence,* 38(3), 352-384. doi:10.1177/0272431616670991 | Children and adolescents (up to 21 yrs) being refugees, asylum seekers or immigrants in the first or second generation | IC1 |
| 97 | Schwartz, S. J., Unger, J. B., Baezconde-Garbanati, L., Zamboanga, B. L., Lorenzo-Blanco, E. I., Des Rosiers, S. E., . . . Szapocznik, J. (2015). Trajectories of cultural stressors and effects on mental health and substance use among Hispanic immigrant adolescents. J*ournal of Adolescent Health*, 56(4), 433-439. doi:10.1016/j.jadohealth.2014.12.011 | Experiences of discrimination or racism | IC2 |
| 98 | Song, R. Z., Fung, J. J., Wong, M. S., & Yao, P. (2020). Attachment as Moderator of Perceived Social-Class Discrimination on Behavioral Outcomes Among Chinese Migrant Children. *Journal of Early Adolescence*, 40(6), 745-771. doi:10.1177/0272431619870604 | Children and adolescents (up to 21 yrs) being refugees, asylum seekers or immigrants in the first or second generation | IC1 |
| 99 | van Dijk, T. K., Agyemang, C., de Witt, M., Hosper, K. (2010). The relationship between perceived discrimination and depressive symptoms among young Turkish-Dutch and Moroccan-Dutch. *European Journal of Public Health*, 21(4), 477-483. doi:10.1093/eurpub/ckq093 | The same sample analyzed in two or more publications | EC4.2 |
| 100 | Verkuyten, M. (2002). Perceptions of ethnic discrimination by minority and majority early adolescents in the Netherlands. *International Journal of Psychology*, 37(6), 321-332. doi:10.1080/00207590244000142 | Outcomes related to the development, well-being or health in children and adolescents | IC3 |
| 101 | Verkuyten, M., & Thijs, J. (2002). Racist victimization among children in The Netherlands: the effect of ethnic group and school. *Ethnic and Racial Studies,* 25(2), 310-331.  doi:10.1080/01419870120109502 | Outcomes related to the development, well-being or health in children and adolescents | IC3 |
| 102 | Verkuyten, M., & Thijs, J. (2006). Ethnic discrimination and global self-worth in early adolescents: The mediating role of ethnic self-esteem*. International Journal of Behavioral Developmen*t, 30(2), 107-116. doi:10.1177/0165025406063573 | Outcomes related to the development, well-being or health in children and adolescents | IC3 |
| 103 | Wang, J.-L., Hsieh, H.-F., Assari, S., Gaskin, J., & Rost, D. H. (2018). The protective effects of social support and engagement coping strategy on the relationship between perceived discrimination and psychological distress among Chinese migrant children. *Youth & Society,* 50(5), 593-614. doi:10.1177/0044118X15619804 | Children and adolescents (up to 21 yrs) being refugees, asylum seekers or immigrants in the first or second generation | IC1 |
| 104 | Xiang, X., Wong, D. F. K., & Hou, K. (2018). The impact of perceived discrimination on personality among Chinese migrant children: The moderating role of parental support. *International Journal of Social Psychiatry,* 64(3), 248-257. doi:10.1177/0020764018758123 | Children and adolescents (up to 21 yrs) being refugees, asylum seekers or immigrants in the first or second generation | IC1 |
| 105 | Xiong, R., Xia, Y., & Li, S. D. (2021). Perceived Discrimination and Aggression Among Chinese Migrant Adolescents: A Moderated Mediation Model. *Frontiers in psychology*, 12, 651270. doi:10.3389/fpsyg.2021.651270 | Children and adolescents (up to 21 yrs) being refugees, asylum seekers or immigrants in the first or second generation | IC1 |
| 106 | Yang, J., Liu, X., Zhao, F., Wang, L., Liu, X., Zhou, H., & Shi, B. (2019). The effects of perceived discrimination and city identity on the social adaptation of migrant children in public and private schools. *Stress and Health: Journal of the International Society for the Investigation of Stress*, 35(3), 341-349. doi:10.1002/smi.2869 | Children and adolescents (up to 21 yrs) being refugees, asylum seekers or immigrants in the first or second generation | IC1 |
| 107 | Ying, Y., & Han, M. (2006). The Effect of Intergenerational Conflict and School-Based Racial Discrimination on Depression and Academic Achievement in Filipino American Adolescents. *Journal of Immigrant & Refugee Studies,* 4(4), 19-35. doi:10.1300/J500v04n04_03 | The same sample analyzed in two or more publications | EC4.2 |

Table 2: Characteristics of primary studies included in the systematic review (k=34).

| **First author (year) [country of authors] {funding}** | **Study design [control / comparison]** | **Countries/regions of origin or ethnicity: % (host country) [migrant or refugee children]** | **% 1. generation / Duration of stay in host countries: M (SD) [Range]** | **Sample size: n (% female)** | **Age in years:  M (SD) [range]** | **Type(s) of discrimination or racism (%, M (SD) in sample) [name/type of instrument; n items]** | **Type(s) of developmental outcomes (%, M (SD) in sample) [name/type of instrument; n items]** |
| --- | --- | --- | --- | --- | --- | --- | --- |
| Adriaanse  et al. (2014) [Netherlands] {government} | cross-sectional survey [Dutch natives] | Morocco: 32, Turkey: 13 (Netherlands) [migrant] | 9 / NA | total: 1,278 (47) IG: 576 (NA) CG: 702 (NA) | total:  12.9 (1.8) [NA]  IG:  Morocco: 12.7 (1.9) [NA]  Turkey: 12.8 (1.7) [NA]  CG: 13.2 (1.7) [NA] | perceived personal discrimination  Morocco (27, NA)  Turkey (26, NA)  CG (8, NA)  [self-report; 3]  perceived group discrimination  Morocco (59, NA)  Turkey (45, NA)  CG (23, NA)  [self-report scale; 3] | SELF-REPORT:  conduct problems  Morocco (23, NA) \| Turkey (20, NA) \| CG (14, NA)  hyperactivity  Morocco (12, NA) \| Turkey (11, NA) \| CG (28, NA)  emotional symptoms  Morocco (12, NA) \| Turkey (11, NA) \| CG (16, NA)  peer problems  Morocco (45, NA) \| Turkey (41, NA) \| CG (28, NA)  pro-social behaviour  Morocco (19, NA) \| Turkey (18, NA) \| CG (23, NA)  total difficulties  Morocco (18, NA) \| Turkey (18, NA) \| CG (19, NA)  TEACHER REPORT:  conduct problems  Morocco (46, NA) \| Turkey (32, NA) \| CG (22, NA)  hyperactivity  Morocco (38, NA) \| Turkey (34, NA) \| CG (26, NA)  emotional symptoms  Morocco (24, NA) \| Turkey (26, NA) \| CG (29, NA)  peer problems  Morocco (31, NA) \| Turkey (22, NA) \| CG (30, NA)  pro-social behaviour  Morocco (23, NA) \| Turkey (16, NA) \| CG (17, NA)  total difficulties  Morocco (37, NA) \| Turkey (29, NA) \| CG (26, NA)  TOTAL DIFFICULTIES (self- or teacher report)  Morocco (44, NA) \| Turkey (40, NA) \| CG (37, NA)  [SDQ; 5 per scale] |
| Astell-Burt  et al. (2012) [UK] {Medical Research  Council} | longitudinal [White adolescents] | India: 17, Pakistan & Bangladesh: 18, Black Caribbean: 31, Nigeria & Ghana: 20, other African: 15 (UK) [migrant] | India (male: 27 \| female: 29), Pakistan & Bangladesh (male: 29 \| female: 18) Black Caribbean (male: 29 \| female: 24), Nigeria & Ghana (male: 24 \| female: 16), other African (male: 73 \| female: 68) / NA | total: 3,409 (46)  IG: 2,536 (47)  CG: 873 (44) | BASELINE:  total: NA [11–13]  IG: NA \| CG: NA  FOLLOW-UP:  total: NA [14–16]  IG: NA \| CG: NA | experienced racism in 3 settings (home, school, living area)  BASELINE:  India (male: 31 \| female: 27, NA)  Pakistan & Bangladesh (male: 35 \| female: 32, NA)  Black Caribbean (male: 24 \| female: 23, NA)  Nigeria & Ghana (male: 29 \| female: 25, NA)  other African (male: 32 \| female: 26, NA)  CG (male: 18 \| female: 14)  FOLLOW-UP:  India (male: 32 \| female: 30, NA)  Pakistan & Bangladesh (male: 27 \| female: 32, NA)  Black Caribbean (male: 29 \| female: 29, NA)  Nigeria & Ghana (male: 39 \| female: 34, NA)  other African (male: 29 \| female: 33, NA)  CG (male: 20 \| female: 17)  [self-report; 3 (1 per setting)] | psychological well-being (total difficulties)  BASELINE:  India (male: NA, 10.6 (NA) \| female: NA, 9.5 (NA))  Pakistan & Bangladesh (male: NA, 10.1 (NA) \| female: NA, 11.4 (NA))  Black Caribbean (male: NA, 10.9 (NA) \| female: NA, 11.5 (NA))  Nigeria & Ghana (male: NA, 9.2 (NA) \| female: NA, 11.3 (NA))  other African (male: NA, 10.7 (NA) \| female: NA, 11.0 (NA))  CG (male: NA, 11.3 (NA) \| female: NA, 11.1 (NA))  FOLLOW-UP:  India (male: NA, 9.8 (NA) \| female: NA, 9.7 (NA))  Pakistan & Bangladesh (male: NA, 9.5 (NA) \| female: NA, 11.0 (NA))  Black Caribbean (male: NA, 9.5 (NA) \| female: NA, 11.0 (NA))  Nigeria & Ghana (male: NA, 8.8 (NA) \| female: NA, 10.9 (NA))  other African (male: NA, 9.5 (NA) \| female: NA, 10.8 (NA))  CG (male: NA, 10.3 (NA) \| female: NA, 11.3 (NA))  [SDQ; 25] |
| Balkaya (2019) [USA] {–} | cross-sectional survey [–] | South-Asia: 71, Middle Eastern / Arab / North Africa: 23, other Asian / bicultural descent: 6 (USA) [migrant] | 11 / 14.1 years (NA) [NA] | 212 (59) | 16.7 (1.6) [13–18] | individual-level religious discrimination (individual-level) (NA, 1.4 (0.6)) [Perceived Discrimination by Adults/Peers Scale (adaptation); NA]  group-level religious discrimination (NA, 3.4 (0.7)) [Perceived Islamophobia Scale; 12] | internalizing problems (NA, 0.6 (0.4))  externalizing problems (NA, 0.4 (0.3))  [YSR; NA] |
| Becerra (2015) [USA] {–} | cross-sectional survey [–] | Latino: 100 (USA) [migrant] | NA | NA | NA | perceived discrimination (NA, 17.2 (4.2)) [parent report (adapted scale); 6] | anger (NA)  worry: short separation (NA)  worry: long separation (NA)  being afraid (NA)  avoiding family activities (NA)  [parent report (scale); 5 (1 each)) |
| Behnke  et al. (2011) [USA] {–} | cross-sectional survey [–] | Mexico: 69, El Salvador: 12, NA: 19 (USA) [migrant] | 16 / NA | 383 (53) | 14.6 (0.6) [14–16] | perceived societal discrimination  male (NA, 1.2 (0.3))  female (NA, 1.3 (0.3))  [self-report scale; 10] | depressive symptoms  male (NA, 1.0 (0.6))  female (NA, 0.8 (0.5))  [CES-D; 20]  global self-esteem  male (NA, 2.8 (0.5))  female (NA, 3.0 (0.5))  [RSES; NA] |
| Beiser (2016) [Canada] {foundations, government, institutes} | cross-sectional survey [–] | Serbia: NA, Vietnam: NA, El Salvador: NA, Ethiopia: NA, Sri Lanka: NA, Afghanistan: NA (Canada) [migrant, refugee] | 100 / NA [≤ 10 years] | total:  478 (NA)  migrant:  326 (51)  refugee:  152 (53) | NA | perceived discrimination by peers and teachers  migrant (NA, 0.9 (NA))  refugee (NA, 1.5 (NA))  [peers: self-report; 10 \| teachers: self-report scale; 1]  feeling welcomed at school  migrant (NA, 31.4 (NA))  refugee (NA, 31.8 (NA))  [self-report scale; 10] | emotional problems  migrant (NA, 10.4 (NA))  refugee (NA, 11.1 (NA))  [self-report symptom scale (adaptation of Ontario Child Health Survey); 8]  aggressive behavior  migrant (NA, 7.2 (NA))  refugee (NA, 7.5 (NA))  [self-report scale; 6] |
| Borges (2011) [USA] {government} | cross-sectional survey [–] | Hispanic/Latino: 19–44, Non-Hispanic/Black/African-American: 40–52, Non-Hispanic/other: 6–18, Non-Hispanic/White: 3–27 (percentages per subsample) (USA) [migrant] | 26 / NA | 1,004 (55–63 per subsample) | NA | nativity-based discrimination (NA) [self-report; 1] | deliberate self-injury (8, NA) [self-report; 1]  suicidal ideation (9, NA) [self-report;1] |
| Cano (2015) [USA] {federal-government research institute} | longitudinal cohort [–] | Cuba: 31, Dominican Republic: 8, Nicaragua: 7, Honduras: 6, Colombia: 6, Mexico: 70, El Salvador: 9, Guatemala: 6 (USA) [migrant] | NA / 2.1 years (1.9) [0–5] | 302 (47) | 14.5 (0.9) [14–17] | perceived ethnic discrimination (NA, 0.8 (0.8)) [self-report scale; 7]  perceived negative context of reception (NA, 8.7 (4.8)) [self-report scale; 6]  bicultural stress (NA, 18.5 (14.4)) [BSS; 20] | depressive symptoms (NA, 30.6 (14.3)) [CES-D; 20]  externalizing behaviour:  substance use (cigarettes: 6, NA \| alcohol: 9, NA) [Monitoring the Future survey (modification); 2]  aggressive behaviour (NA, 5.4 (6.2)) [YSR (subscale); 17]  rule-breaking behavior (NA, 4.1 (5.2)) [YSR (subscale); 15] |
| Chun (2011) [South Korea] {–} | cross-sectional survey [Korean children] | Japan: 44, China: 37, Vietnam: 8, Philippines: 7, Indonesia: 3, Taiwan: 1, Russia: 1 (South Korea) [migrant] | NA | total: 207 (NA) IG: 105 (NA) CG: 102 (NA) | NA | perceived discrimination  IG (NA, 16.4 (6.2))  CG (NA, 15.0 (4.9))  [Everyday Discrimination Scale; 10] | depressive symptoms  IG (NA, 6.1 (4.9))  CG (NA, 5.1 (4.1))  [K-YSR (subscale); 14]  ego resiliency  IG (NA, 39.0 (6.9))  CG (NA, 42.1 (6.5))  [Ego Resiliency Scale; NA]  teacher-student relationships  IG (36.2 (6.0))  CG (38.7 (6.7))  [self-assessment scale; 10] |
| Correa-Velez (2010) [Australia] {foundations, university} | longitudinal [–] | Africa (Sudan, Ethiopia, Liberia, Uganda): 68, Middle East (Iraq, Afghanistan, Iran, Kuwait): 27, Eastern Europe (Bosnia and Croatia) and Southeast Asia (Burma): 5 (Australia) [refugee] | 100 / 5.3 months (4.3) [0–16] | 97 (49) | 15.1 (1.6) [11–19] | perceived discrimination (21, NA) [Experiences of Discrimination Scale (subset); 1] | well-being  physical (NA, 79.9 (13.0))  psychological (NA, 77.6 (14.5))  social relationships (NA, 85.0 (16.8))  environment (NA, 73.8 (14.3))  [WHOQOL-BREF; 26]  subjective health status (NA, 80.6 (21.0)) [self-report; 1]  happiness (NA, 84.7 (18.4)) [self-report; 1] |
| Cristini et al. (2011) [Italy] {–} | cross-sectional [–] | Romania: 24, Moldavia: 17, Albania: 16, Morocco: 12, other: 31 (Italy) [migrant] | 100 / NA | 214 (33) | 17.6 (1.6) [NA] | perceived discrimination (NA, 2.4 (0.8)) [Acculturative Stress Inventory for Children (adapted subscale); 8] | depressive symptoms (NA, 1.7 (0.6)) [CES-D (Italian version, adapted); 16] |
| D’hondt (2016) [Belgium] {university} | cross-sectional [–] | Turkey: 40, Morocco: 60 (Belgium) [migrant] | NA | 553 (50) | 16.0 (NA) [NA] | teacher ethnic discrimination (34, NA) [self-report & self-report scale; 6] | school misconduct (NA, 30.1 (9.8)) [self-report scale; 17] |
| el Bouhaddani (2019) [Netherlands] {research funding organization (ZonMw)} | cross-sectional [Dutch youth] | Morocco: 13, Turkey: 11, Suriname: 4, Antilles: 3 (Netherlands) [migrant] | 91 / NA | total: 1,194 (NA)  IG: 371 (NA)  CG: 823 (53) | total: 13.7 (0.6) [NA]  IG: NA  CG: 13.5 (0.6) [NA] | perceived personal discrimination  Morocco (23, NA) \| Turkey (9, NA) \| Suriname (25, NA) \| Antilles (23, NA) \| CG (4, NA)  perceived group discrimination Morocco (74, NA) \| Turkey (66, NA) \| Suriname (74, NA) \| Antilles (64, NA) \| CG (28, NA)  [self-report questionnaire; personal: 3 \| group: 4] | psychotic experiences  overall (Morocco: 20, NA \| Turkey: 13, NA \| Suriname: 15, NA \| Antilles: 10, NA \| CG: 17, NA)  hallucination (Morocco: 11, NA \| Turkey: 6, NA \| Suriname: 11, NA \| Antilles: 10, NA \| CG: 12, NA)  delusion (Morocco: 14, NA \| Turkey: 9, NA \| Suriname: 8, NA \| Antilles: 3, NA \| CG: 10, NA)  [PQ-16 (subset); 14] |
| Ellis (2008) [USA] {federal-government research institute} | cross-sectional survey [–] | Somalia: 100 (USA) [refugee] | 53 / 5.4 years (3.3) [1–14] | 135 (38) | 15.4 (2.2) [11–20] | perceived discrimination (NA) [Every Day Discrimination questionnaire; 9] | PTSD (NA) [UCLA PTSD–I; 22]  Depression (NA) [DSRS; 18] |
| Espinosa (2020) [USA] {–} | longitudinal [–] | White: 14, Black: 7, Asian: 30, Hispanic: 33, multiracial: 16  (USA) [migrant] | 0 / – | 4,288 (52) | 17.2 (0.9) [15–21] | perceived discrimination  Wave 1 (NA, 0.5 (0.5))  Wave 2 (NA, 0.6 (0.5))  [self-report; NA] | depression symptoms  Wave 1 (NA, 1.6 (0.6))  Wave 2 (NA, 1.7 (0.6))  [CES-D (short form); NA]  self-esteem  Wave 1 (NA, 3.3 (0.5))  Wave 2 (NA, 3.4 (0.5))  [RSES; 10] |
| Guerra (2019) [Portugal] {European Fund for the Integration of Third Country Nationals} | cross-sectional survey [Portuguese youth] | Africa: NA, America: NA, Asia: NA, Europe: NA (Portugal) [migrant] | 54 / NA | total: 593 (55)  IG: 425 (NA)  CG: 168 (NA) | total: 10.7 (1.4) [NA]  IG: NA  CG: NA | perceived discrimination  immigrant (NA, 2.6 (1.0))  immigrant descendant (NA, 2.3 (0.9))  native CG (NA, 2.3 (0.8))  [self-report scale; 4] | social-emotional well-being  feelings (immigrant: NA, 4.0 (0.9) \| immigrant descendant: NA, 4.1 (0.7) \| native CG: NA, 4.1 (0.8))  school (immigrant: NA, 4.1 (0.9) \| immigrant descendant: NA, 3.9 (0.8) \| native CG: NA, 4.0 (0.9))  [KIDSCREEN-52; NA]  school achievement  immigrant (–, 2.9 (0.6))  immigrant descendant (–, 3.0 (0.5))  native CG (–, 3.2 (0.6))  [averaged 1st & 2nd term final grades; –] |
| Kauff (2017) [Germany] {NORFACE ERA NET Plus Migration in  Europe-programme} | cross-sectional & longitudinal survey [–] | NA (Germany, Netherlands, Sweden)  [migrant] | NA | Wave 1: 4,334 (NA)  Wave 2: 3,236 (NA) | Wave 1:  15.0 (NA) [14]  Wave 2:  16.0 (NA) [NA] | ethnic victimization in school class (21, 0.3 (0.7)) [network data by peer nomination procedure; –]  perceived discrimination:  public transportation (12, 1.1 (0.4)) \| leisure services (13, 1.2 (0.4)) \| police/security (17, 1.3 (0.6)) [self-report scale; 3] | health problems  Wave 1 (NA, 2.5 (0.8))  Wave 2 (NA, 2.6 (0.9))  [self-report scale; 3] |
| Kiang (2015) [USA] {university} | cross-sectional [–] | Hmong: 28, multi-ethnic: 22, South Asia: 11, China: 8, pan-ethnic: 8, others: 23 (USA) [migrant] | 26 / NA | 159 (60) | 9th grade (48%): 14.4 (0.6) [NA]  10th grade (52%):  15.6 (0.7) [NA] | perceived discrimination (NA) [self-report scale; 7] | perceived academic performance (NA) [self-report scale adapted from Eccles (1983); 2]  positive school value (NA) [self-report scale adapted from Eccles (1983); 4]  positive relationships with others (NA) [positive relationships subscale (psychological well-being measure; Ryff, 1989); 9]  self-esteem (NA) [RSES; 10]  depressive symptoms (NA) [CES-D-10; 10] |
| Kogan (2019)  [Georgia] {–} | cross-sectional [–] | Ethiopia: 100 (Israel) [migrant] | 15 / NA | 110 (60) | 17.0 (1.0) [15–18] | racial discrimination (NA, 1.1 (0.2)) [Experiences of Discrimination and Racism Scale; 18] | delinquency (NA) [National Youth Survey (subset); 18] |
| Liebkind  (2000) [Finland] {foundation, academy} | comparative  cross-sectional [–] | Russia: 29, Vietnam: 46, Turkey: 15, Somalia: 10 (Finland)  [migrant] | 90 / 6.3 years (4.8) [NA] | 588 (49) | 15.3 (2.0) [11–20] | perceived discrimination  Russia (NA, 2.3 (NA))  Turkey (NA, 1.9 (NA))  Vietnam (NA, 2.2 (NA))  Somalia (NA, 2.7 (NA))  [self-report scale; 9] | acculturative stress  Russia (NA, 2.2 (NA))  Turkey (NA, 2.2 (NA))  Vietnam (NA, 2.3 (NA))  Somalia (NA, 2.0 (NA))  [adapted scale (subscales: anxiety, depression, psychosomatic symptoms); 15]  behavioural problems  Russia (NA, 1.6 (NA))  Turkey (NA, 1.4 (NA))  Vietnam (NA, 1.3 (NA))  Somalia (NA, 1.4 (NA))  [self-report scale (adaptation from Olweus, 1989); 10]  self-esteem  Russia (NA, 3.7 (NA))  Turkey (NA, 3.8 (NA))  Vietnam (NA, 3.5 (NA))  Somalia (NA, 3.7 (NA))  [RSES; 10]  life satisfaction  Russia (NA, 3.4 (NA))  Turkey (NA, 3.7 (NA))  Vietnam (NA, 3.5 (NA))  Somalia (NA, 4.0 (NA))  [Satisfaction With Life Scale; 5]  sense of mastery  Russia (NA, 4.1 (NA))  Turkey (NA, 4.0 (NA))  Vietnam (NA, 3.7 (NA))  Somalia (NA, 3.5 (NA))  [self-report scale (adapted); NA] |
| Neto (2000)  [Portugal] {–} | cross-sectional [Portuguese youth residing in Portugal] | Portugal: 100 (Switzerland)  [migrant] | 93 / 7.2 (4.1) [NA] | total: 458 (NA)  IG: 95 (67)  CG: 363 (47) | total: NA  IG: 16.1 (1.4) [14–19]  CG: 14.5 (1.4) [NA] | perceived discrimination (NA, 16.9 (7.7) [self-report scale; 7] | loneliness  IG (NA, 9.6 (3.4))  CG (NA, 10.0 (NA))  [Revised UCLA Loneliness Scale (brief Portuguese version); 6]  stressful experience adaptation (NA, 25.5 (7.5)) [f Social Situations Questionnaire (adaptation); 13]  self-esteem (NA, 36.9 (5.8)) [RSES; 10]  mastery (NA, 23.7 (3.7))  [self-report scale (adapted from existing scales), 6]  depressive, anxiety & psychosomatic symptoms (NA, 38.0 (9.8)) [self-report scale (adapted from existing scales); 15 (5 per subscale)] |
| Neto (2001)  [Portugal] {–} | cross-sectional [–] | Angola: 35, Cape Verde: 36, India: 30 (Portugal) [migrant] | 35 / 6.9 years (4.5) [3–11] | 313 (66) | 15.0 (1.9) [NA] | perceived discrimination (NA, 19.6 (7.0)) [self-report scale; 7] | in-group social interaction (NA, 12.8 (4.5)) [self-report scale; 4]  out-group social interaction (NA, 15.6 (3.1)) [self-report scale; 4]  stressful experience acculturation (NA, 26.5 (8.5)) [Social Situations Questionnaire (adaptation); 13]  mastery (NA, 23.5 (4.3)) [self-report scale (adapted); 6]    self-esteem (NA, 37.4 (6.1)) [RSES; 10]  depressive, anxiety & psychosomatic symptoms (NA, 34.7 (9.6)) [self-report scale (adapted); 15 (5 per subscale)]  satisfaction with life (NA, 18.0 (4.2)) [Satisfaction with Life Scale; 5] |
| Oczlon (2021)  [Austria] {–} | cross-sectional [–] | NA (Austria)  [migrant] | 40 / NA | 700 (45) | 12.6 (1.1) [10–16] | perceived discrimination (NA, 1.8 (1.1)) [self-report scale (adapted); 3] | self-esteem (NA, 4.0 (0.7)) [RSES (German adaptation, subset); 3]  academic self-concept (NA, 3.6 (0.7)) [academic self-concept scale (SESSKO); 5]  academic achievement (–, 3.1 (0.8)) [averaged recent report card grades in three subjects; –] |
| Okamoto  et al. (2009) [USA] {federal-government research institute} | cross-sectional [–] | Central America: NA, South America: NA, Mexico: NA, other: NA (USA) [migrant] | 15 / NA | 1,332 (51) | 14.0 (0.4) [13–16] | perceived discrimination (NA, 1.7 (0.6)) [self-report scale; 10] | substance use  cigarettes (lifetime: 26, NA \| past 30 days: 7, NA)  alcohol: (lifetime: 48, NA \| past 30 days: 25, NA)  binge drinking (lifetime: – \| past 30 days: 13, NA)  marijuana (lifetime: 19, NA \| past 30 days: 11, NA)  inhalants (lifetime: 12, NA \| past 30 days: 8, NA)  [self-report; NA] |
| Oppedal (2004)  [Norway] {–} | longitudinal [–] | Pakistan: 37, Turkey: 8, Vietnam: 8, other Asian countries: 21, Africa: 10, Eastern Europe: 9 (Norway) [migrant] | 44 / 6.9 years (4.1) [NA] | Wave 1:  160 (NA)  Wave 2:  137 (63) | Wave 1:  NA [ca. 13]  Wave 2:  NA [ca. 14] | perceived discrimination in class  Wave 1 (NA, 1.4 (0.5))  Wave 2 (NA, 1.4 (0.6))  [self-report scale; 2] | mental health  Wave 1 (NA, 1.5 (0.4))  Wave 2 (NA, 1.5 (0.4))  [HSCL-25 (subset); 23]  global self-esteem  Wave 1 (NA, 3.1 (0.5))  Wave 2 (NA, 3.1 (0.5))  [RSES; 10] |
| Oppedal (2011)  [Norway] {–} | cross-sectional [–] | Sample 1 (adolescents):  Turkey: 36, Somalia: 31, Vietnam: 33  Sample 2 (pre-adolescents):  Turkey: 28, Somalia: 22, Sri Lanka: 50  (Norway) [migrant] | Sample 1 (adolescents):  65 / NA  Sample 2 (pre-adolescents):  22 / NA | Sample 1 (adolescents):  287 (50)  Sample 2 (pre-adolescents): 359 (48) | Sample 1 (adolescents):  NA [15–16]  Sample 2 (pre-adolescents):  NA [10–13] | perceived discrimination  Sample 1 (adolescents):  Turkey (NA, 1.8 (0.7))  Somalia (NA, 2.0 (0.7))  Vietnam (NA, 1.8 (0.6))  Sample 2 (pre-adolescents):  Turkey (NA, 1.6 (0.5))  Somalia (NA, 1.6 (0.7))  Sri Lanka (NA, 1.7 (0.7))  [self-report scale; 5] | internalizing problems  Sample 1 (adolescents):  Turkey (NA, 1.4 (0.5))  Somalia (NA, 1.3 (0.4))  Vietnam (NA, 1.7 (0.6))  [HSCL; 10]  Sample 2 (pre-adolescents):  Turkey (NA, 3.2 (2.1))  Somalia (NA, 2.8 (2.3))  Sri Lanka (NA, 3.5 (2.4))  [SDQ (Emotional Symptoms subscale); 5] |
| Oxman-Martinez  et al. (2012)  [Canada] {foundations, government, institutes} | cross-sectional [–] | China: 38, Hong Kong: 36, Philippines: 26 (Canada) [migrant] | NA / 4.7 years (NA) [NA] | 1,053 (50) | 11.9 (NA) [11–13] | perceived peer discrimination (33, 1.0 (1.9)) [self-rating scale; 2]  perceived teacher discrimination (NA, 1.0 (1.7)) [self-report scale; 2] | sense of competence in peer relationships (NA) [Social Competence Scale (Beiser et al., 1993); 4]  self-esteem (NA) [Self-Description Questionnaire (General-Self Scale); 4]  perception of academic competence (NA) [self-rating scale; 1]  factual academic grades (NA) [–] |
| Özdemir (2013) [Sweden] {government} | longitudinal survey [–] | 54 countries in the Middle East, East Africa, Asia, South America, parts of former Yugoslavia (Sweden) [migrant] | 38 / NA [> 5 years in 69% of sample] | 330 (49) | 14.1 (0.9) [12–17] | ethnic harassment (80, 1.5 (0.5)) [self-report scale; 6] | depressive symptoms (T1: NA, 1.6 (0.6) \| T2: NA, 1.8 (0.7)) [CES-DC; 16]  self-esteem (T1: NA, 3.2 (0.6) \| T2: NA, 3.1 (0.6)) [RSES; 10]  positive relationships with teachers at T1 (NA, 3.0 (0.7)) [self-report scale; 6]  perceived academic failure (T1: NA, 1.5 (0.6) \| T2: NA, 1.6 (0.6)) [self-report scale; 4] |
| Pantzer (2006) [Spain] {government, institute} | cross-sectional [Spanish youth] | Maghreb: 5, Latino: 40, Western Europe: 14, Eastern Europe: 32, Asia: 7, Africa: 1 (Spain) [migrant] | 100 / NA | Total: 1,235 (51)  IG: 226 (NA)  CG: 1,009 (NA) | total: NA [12–18]  IG: NA  CG: NA | perceived discrimination  IG (NA, 6.5 (2.1))  CG (NA, 5.9 (2.0))  [self-report (adapted); 5] | health-related quality of life  IG (NA, 60.8 (13.1))  CG (NA, 65.0 (12.5))  [VSP-A (Spanish version); 39]  psychological distress  IG (NA, 2.6 (2.8))  CG (NA, 2.2 (2.0))  [GHQ-12 (Spanish version); 12] |
| Potochnick (2010)  [USA] {foundation} | cross-sectional [–] | Mexico: 70 (USA) [migrant] | 100 / NA | 255 (NA) | NA [12–19] | experienced discrimination (42, NA) [self-report; 1]  perceived discrimination (NA, 2.6 (0.1)) [Youth Adaptation and Growth Questionnaire (adaptation); 4] | depression (7, 7.8 (NA)) [CDI; 27]  anxiety (29, 50.2 (NA))  [MASC-10; 10] |
| Sabatier (2008)  [Canada] {institute, federal-government research institute} | cross-sectional [–] | Algeria: 23, Antilles: 16, Morocco: 25, Portugal: 24, Vietnam: 13 (France)  Greece: 27, Haiti: 29, Italy: 26, Vietnam: 18 (Canada)  [migrant] | 0 / – | 718 (53) | 15.5 (1.8) [11–19] | perceived discrimination (personal & group)  France (NA, 2.1 (0.9))  Canada (NA, 2.0 (0.8))  [self-report scale; 12 (6 each)] | deviance  France (NA, 1.4 (0.4))  Canada (NA, 1.3 (0.3))  [self-report scale; 11]  general self-esteem  France (NA, 3.1 (0.5))  Canada (NA, 3.3 (0.5))  [RSES; 10]  domain-specific self-esteem  familial self-esteem (NA)  social self- esteem (France: 3.8 (0.6) \| Canada: 4.7 (0.8))  school self-esteem (France: 3.5 (0.8) \| Canada: 5.2 (0.6))  emotional self-esteem (France: 3.7 (0.6) \| Canada: 4.6 (0.8))  [France: Coopersmith’s Self-Esteem Inventory; NA \|  Canada: Offer Self-Image Questionnaire; NA]  stress symptoms  France (NA, 2.1 (0.6))  Canada: –  [self-report scale; 28] |
| Tummala-Narra (2013) [USA] {college} | cross-sectional [–] | NA (USA) [migrant] | 52 / NA | 95 (46) | 15.1 (1.1) [13–19] | adult discrimination (NA, 0.1 (0.2)) [self-report scale (adapted); 21]  peer discrimination (NA, 0.2, (0.2)) [self-report scale (adapted; 21] | depressive symptoms (NA, 0.9 (0.5))  [CES-DC; 20] |
| Verkuyten (1998)  [Netherlands] {–} | cross-sectional [–] | Turkey: 58, Morocco: 43 (Netherlands) [migrant] | NA | 170 (55) | 13.7 (NA) [12–15] | perceived personal discrimination  Turkey (NA, 0.9 (0.9))  Morocco (NA, 1.2 (0.9))  [self-report scale; 3]  perceived group discrimination Turkey (NA, 1.8 (0.8))  Morocco (NA, 2.2 (1.2))  [self-report scale; 3] | ethnic self-esteem  Turkey (NA, 3.7 (0.7))  Morocco (–)  [self-report scale; 5]  personal self-esteem  Turkey (NA, 2.5 (0.6))  Morocco (NA, 2.6 (0.7))  [PCSC (subset); 5]  perceived competence  social (Turkey: NA, 3.1 (0.6) \|  Morocco (NA, 2.9 (0.8))  cognitive (Turkey: NA, 2.8 (0.6) \| Morocco (NA, 2.8 (0.7))  physical (Turkey: NA, 2.7 (0.7) \| Morocco (NA, 2.9 (0.8))  [PCSC; 6]  sense of control  Turkey (–)  Morocco (NA, 2.8 (0.8))  [Locus of Control Scale for Children (subset); 6] |
| Verkuyten (2004) [Netherlands] {–} | Study 1 & 2:  cross-sectional [–] | Study 1 & 2:  Turkey: 100 (Netherlands) [NA] | Study 1 & 2:  NA | Study 1:  161 (49)  Study 2:  112 (53) | Study 1:  14.7 (NA) [13–16]  Study 2:  11.6 (NA) [11–12] | perceived discrimination  Study 1 (NA, 1.5 (0.7))  Study 2 (NA, 2.3 (0.8))  [self-report scale; Study 1: 1 \| Study 2: 3] | educational performance  Study 1 (NA, 5.7 (1.3))  Study 2 (NA, 6.9 (1.7))  [Study 1: Willig scale & grade in the weakest area; 2 \| Study 2: Willig scale; 3]  global self-worth  Study 1 (NA, 3.7 (0.6))  Study 2 (NA, 3.9 (0.7))  [Study 1: RSES; NA \| Study 2: RSES (subset); 6]  academic self-esteem  Study 1 (NA, 3.3 (0.6))  Study 2 (NA, 3.7 (0.6))  [Study 1: Self-Perception Profile for Adolescents (scholastic competence subscale); NA \| Study 2: PCSC; NA] |

*Notes*. CG = control/comparison group, IG = index group, NA = not available, UK = United Kingdom, USA = United States of America; BSS = Bicultural Stress Scale, CDI = Children’s Depression Inventory, CES-D(C) = Center for Epidemiological Studies Depression Scale (for Children), DSRS = Depression Self-Rating Scale, GHQ-12 = Goldberg’s general health questionnaire, HSCL-25 = The Hopkin’s Symptom Checklist, K-YSR = Korean Youth Self Report, MASC-10 = Multidimensional Anxiety Scale for Children, PCSC = Perceived Competence Scale for Children, PQ-16 = Prodromal Questionnaire, RSES = Rosenberg Self-Esteem Scale, SESSKO = academic self-concept scale [German: Skalen zur Erfassung des schulischen Selbstkonzepts], SDQ = Strengths and Difficulties Questionnaire, UCLA PTSD-I = UCLA Posttraumatic Stress Disorder Index, VSP-A = Vecu et Sante Percue de l’Adolescent, WHOQOL-BREF = World Health Organization Quality of Life-Bref, YSR = Youth Self-Report; reported percentages were rounded to whole numbers, reported results were shortened to two decimal points.

Table 3: Reported results for the association between discrimination or racism and developmental outcomes in the included studies (k=34)

| **First author (year)** | **Type(s) of analysis** | **Assessed association(s)** |  | **Strength of association** | | | | | | |
| --- | --- | --- | --- | --- | --- | --- | --- | --- | --- | --- |
|  |  |  |  | ***r*** | ***B (SE)*** | ***β*** | ***OR [CI]*** | ***other*** | ***p*** |  |
| **MENTAL AND PHYSICAL HEALTH-RELATED OUTCOMES (k=30)** | | | | | | | | | | |
| ***SELF-ESTEEM (k=12)*** | | | | | | | | | | |
| Behnke (2011) | correlation, SEM | PD (societal) – global self-esteem |  | -.17 | – | NA | – | – | cor: < .05  SEM: NA | * |
| Espinosa (2020) | correlation | PD (W1) – self-esteem |  | W1: -.11  W2: -.09 | – | – | – | – | W1: < .001  W2: < .001 | ***  *** |
|  |  | PD (W2) – self-esteem |  | W1: -.03  W2: -.08 | – | – | – | – | W1: > .05  W2: < .001 | *** |
|  | multigroup path analysis | PD – self-esteem (W2) |  |  | W1: -.24 (.15)  W2: -.67 (.16) |  |  |  | W1: > .05  W2: < .001 | *** |
| Kiang (2015) | hierarchical linear model | PD – self-esteem |  | – | -.18 (.04) | – | – | – | < .001 | *** |
| Liebkind (2000) | correlation, regression | discrimination – self-esteem |  | -.29 | – | -.24 | – | – | cor: < .01  reg: < .001 | **  *** |
| Neto (2000) | correlation | PD – self-esteem |  | -.21 | – | – | – | – | < .05 | * |
| Oczlon (2021) | correlation | PD – self-esteem |  | -.14 | – | – | – | – | < .05 | * |
| Oppedal (2004) | correlation, SEM | PD in class – global self-esteem (longitudinal change) |  | cor: -.23  SEM: -.16 | – | – | – | – | cor: < .01  SEM: < .05 | **  * |
| Oxman-Martinez  et al. (2012) | correlation, regression | peer discrimination – self-esteem |  | -.11 | -.09 (.05) | -.07 | – | – | cor: < .01  reg: > .05 | ** |
|  |  | teacher discrimination – self-esteem |  | -.16 | -.15 (.05) | -.10 | – | – | cor: < .01  reg: < .01 | **  ** |
| Özdemir (2013) | correlation, mediated regression | ethnic harassment (T1) – self-esteem |  | T1: -.21  T2: -.32 | – | T1: –  T2: -.25 | – | – | cor (T1, T2):  < .001  reg: < .001 | ***  *** |
| Sabatier (2008) | multiple hierarchical regression | PD – self-esteem (global) |  | – | – | FR: NA  CA: -.19 | – | – | FR: NA  CA: < .01 | ** |
|  |  | PD – self-esteem (familial) |  | – | – | NA | – | – | NA |  |
|  |  | PD – self-esteem (school) |  | – | – | NA | – | – | NA |  |
|  |  | PD – self-esteem (social) |  | – | – | NA | – | – | NA |  |
|  |  | PD – self-esteem (emotional) |  | – | – | FR: NA  CA: -.16 | – | – | FR: NA  CA: < .05 | * |
| Verkuyten (1998) | correlation | PD (personal) – self-esteem (ethnic) |  | TR: -.09  MA: – | – | – | – | *–* | TR: > .05  MA: – |  |
|  |  | PD (personal) – self-esteem (personal) |  | TR: -.31  MA: -.33 | – | – | – | *–* | TR: < .01  MA: < .01 | **  ** |
|  |  | PD (group) – self-esteem (ethnic) |  | TR: -.39  MA: – | – | – | – | – | TR: < .01  MA: – | ** |
|  |  | PD (group) – self-esteem (personal) |  | TR: -.10  MA: -.03 | – | – | – | – | TR: > .05  MA: > .05 |  |
| Verkuyten (2004) | correlation | PD – self-esteem (academic) |  | S1: -.05  S2: -.04 | – | – | – | – | S1: > .05  S2: > .05 |  |
|  | multiple regression | PD – self-esteem (academic) |  | – | – | S1: -.06  S2: -.03 | – | – | S1: > .05  S2: > .05 |  |
| ***DEPRESSIVE SYMPTOMS (k=10)*** | | | | | | | | | | |
| Behnke et al. (2011) | correlation, SEM | PD (societal) – depressive symptoms |  | .30 | – | m: .10  f: .21 | – | – | cor: < .05  SEM (m&f):  < .05 | *  * |
| Cano (2015) | correlation | perceived negative context of reception – depressive symptoms |  | T1: .38  T2: .25 | – | – | – | – | T1: < .01  T2: < .01 | **  ** |
|  |  | bicultural stress – depressive symptoms |  | T1: .45  T2: .36 | – | – | – | – | T1: < .01  T2: < .01 | **  ** |
|  |  | PD – depressive symptoms |  | T1: .30  T2: .29 | – | – | – | – | T1: < .01  T2: < .01 | **  ** |
|  | path analysis | cultural stress^a^ – depressive symptoms |  | – | – | .38 | – | – | < .01 | ** |
| Chun (2011) | path analysis | PD – depression |  | – | – | IG: .40  CG: .32 | – | – | IG: < .001  CG: < .001 | ***  *** |
| Cristini et al.  (2011) | correlation, regression | PD – depressive symptoms |  | .24 | .19 (.06) | – | – | – | cor: < .01  reg: < .01 | **  ** |
| Ellis (2008) | correlation, multiple linear regression | PD – depression |  | .42 | .07 (.03) | .24 | – | – | cor: < .01  reg: < .01 | **  ** |
| Espinosa (2020) | correlation | PD (W1) – depression |  | W1: .15  W2: .10 | – | – | – | – | W1: < .001  W2: < .001 | ***  *** |
|  |  | PD (W2) – depression |  | W1: .08  W2: .12 | – | – | – | – | W1: < .001  W2: < .001 | ***  *** |
|  | multigroup path analysis | PD – depression (W2) |  | – | W1: .02 (.02)  W2: .09 (.02) | – | – | – | W1: > .05  W2: < .001 | *** |
| Kiang (2015) | hierarchical linear model | PD – depressive symptoms |  | – | .22 (.03) | – | – | – | < .001 | *** |
| Özdemir (2013) | correlation, mediated regression | ethnic harassment (T1) – depression |  | T1: .24  T2: .21 | – | T1: –  T2: .12 | – | – | cor (T1, T2):  < .001  reg: < .05 | ***  * |
| Potochnick (2010) | logit regression | ED – depression |  | – | – | – | 1.33 [0.17–10.48] | – | > .10 |  |
|  |  | PD – depression |  | – | – | – | 4.09 [0.69–24.43] | – | > .10 |  |
| Tummala-Narra (2013) | multiple regression | PD (adult) – depressive symptoms |  | – | 1.88 (.59) | – | – | *–* | < .01 | ** |
|  |  | PD (peer) – depressive symptoms |  | – | 1.38 (.38) | – | – | *–* | < .01 | ** |
| ***EXTERMALIZING PROBLEMS (k=5)*** | | | | | | | | | | |
| Balkaya (2019) | correlation, SEM | religious discrimination (individual) – externalizing problems |  | .13 | – | .14 | – | – | cor: > .05  SEM: < .05 | * |
|  |  | religious discrimination (group) – externalizing problems |  | .02 | – | NA | – | – | cor: > .05  SEM: NA |  |
| Becerra (2015) | ordinal logistic regression | PD – anger |  | – | .04 (.10) | – | 1.04 [NA] | – | > .05 |  |
| Beiser (2016) | regression | PD – aggressive behaviour |  | – | – | .31 | – | – | < .001 | *** |
|  |  | feeling welcomed at school – aggressive behaviour |  | – | – | -.05 | – | – | < .01 | ** |
| Cano (2015) | correlation | perceived negative context of reception – aggressive behaviour |  | T1: .22  T2: .15 | – | – | – | – | T1: < .01  T2: < .05 | **  * |
|  |  | bicultural stress – aggressive behaviour |  | T1: .56  T2: .40 | – | – | – | – | T1: < .01  T2: < .01 | **  ** |
|  |  | PD – aggressive behaviour |  | T1: .30  T2: .34 | – | – | – | – | T1: < .01  T2: < .01 | **  ** |
|  | path analysis | cultural stress^a^ – aggressive behaviour |  | – | – | .28 | – | – | < .001 | *** |
| Liebkind (2000) | correlation, regression | discrimination – behavioural problems |  | .19 | – | .21 | – | – | cor: < .01  reg: < .001 | **  *** |
| ***INTERNALIZING PROBLEMS (k=4)*** | | | | | | | | | | |
| Balkaya (2019) | correlation, SEM | religious discrimination (individual) – internalizing problems |  | .16 | – | .13 | – | – | cor: < .05  SEM: < .05 | *  * |
|  |  | religious discrimination (group) – internalizing problems |  | .10 | – | NA | – | – | cor: > .05  SEM: NA |  |
| Becerra (2015) | ordinal logistic regression | PD – worry (short separation) |  | – | .05 (.03) | – | 1.05 [NA] | – | > .05 |  |
|  |  | PD – worry (long separation) |  | – | .20 (.14) | – | 1.22 [NA] | – | > .05 |  |
|  |  | PD – being afraid |  | – | .24 (.12) | – | 1.27 [NA] | – | < .05 | * |
| Beiser (2016) | regression | PD – emotional problems |  | – | – | .40 | – | – | < .001 | *** |
|  |  | feeling welcomed at school – emotional problems |  | – | – | -.04 | – | – | > .05 |  |
| Oppedal (2011) | correlation | PD – internalizing problems (Sample 1: adolescents) |  | TR: .22  SO: .09  VN: .22 | – | – | – | – | TR: < .05  SO: > .05  VN: < .05 | *  * |
|  |  | PD – internalizing problems (Sample 2: pre-adolescents) |  | TR: .34  SO: .40  LK: .51 | – | – | – | – | TR: < .01  SO: < .01  LK: < .001 | **  **  *** |
|  | hierarchical regression | PD – internalizing problems (Sample 1: adolescents) |  | – | – | TR: .15  SO: .23  VN: .15 | – | – | TR: > .05  SO: < .01  VN: > .05 | ** |
|  |  | PD – internalizing problems (Sample 2: pre-adolescents) |  | – | – | TR: .19  SO: .31  LK: .54 | – | – | TR: > .05  SO: < .01  LK: < .001 | **  *** |
| ***GLOBAL (MENTAL) HEALTH (k=4)*** | | | | | | | | | | |
| Adriaanse  et al.(2014) | logistic regression | PD (personal) – high mental health problems |  | – | – | – | 1.83 [1.32–2.55] | – | < .001 | *** |
|  |  | PD (group, low) – high mental health problems |  | – | – | – | 1.00 [–] | – | – |  |
|  |  | PD (group, medium) – high mental health problems |  | – | – | – | 1.19 [0.78–1.81] | – | > .05 |  |
|  |  | PD (group, high) – high mental health problems |  | – | – | – | 1.71 [1.30–2.24] | – | < .001 | *** |
| Correa-Velez (2010) | generalized estimating equations | ED – subjective health status |  |  |  |  | NA | NA | > .05 |  |
| Kauff (2017) | multilevel modelling | victimization in school class – health problems |  | – | cross: .05 (.01)  long: 0.3 (0.1) | – | – | – | cross: < .001  long: < .01 | ***  ** |
|  |  | PD in public transportation – health problems |  | – | cross: .03 (.02)  long: .002 (0.2) | – | – | – | cross: < .05  long: > .05 | * |
|  |  | PD in leisure services – health problems |  | – | cross: .03 (.02)  long: -.01 (.02) | – | – | – | cross: < .05  long: > .05 | * |
|  |  | PD by police/security – health problems |  | – | cross: .10 (.01)  long: .04 (.02) | – | – | – | cross: < .001  long: < .01 | ***  ** |
| Oppedal (2004) | correlation, SEM | PD in class – mental health (longitudinal change) |  | cor: .24 | – | – | – | SEM: *t* = 1.90 | cor: < .01  SEM: > .05 | ** |
| ***WELL-BEING (k=3)*** | | | | | | | | | | |
| Astell-Burt et al. (2012) | logit regression | experienced racism – psychological well-being |  | – | – | – | IN:  – [10.83–12.04]  PK/BD:  – [10.82–11.99]  BC:  – [11.30–12.23]  NG/GH:  – [10.41–11.50]  oA:  – [11.12–12.33]  CG:  – [11.86–12.89] | mean total difficulties:  IN: 11.44  PK/BD: 11.40  BC: 11.76  NG/GH: 10.96  oA: 11.72  CG: 12.37 | IN:  < .001  PD/BD:  < .001  BC:  < .001  NG / GH:  < .001  oA:  < .001  CG:  < .001 | ***  ***  ***  ***  ***  *** |
| Correa-Velez (2010) | generalized estimating equations | ED – well-being (physical) |  | – | – | – | – [-8.00– -1.04] | mean change:  -4.52 | .011 | * |
|  |  | ED – well-being (psychological) |  |  |  |  | NA | NA | > .05 |  |
|  |  | ED – well-being (social relationships) |  |  |  |  | NA | NA | > .05 |  |
|  |  | ED – well-being (environment) |  | – | – | – | – [-9.18– -2.22] | mean change:  -5.70 | .001 | ** |
| Guerra (2019) | correlation | PD – well-being (feelings) |  | IM: -.14  ID: -.08  CG: -.12 | – | – | – | – | IM: < .05  ID: > .05  CG: > .05 | * |
|  |  | PD – well-being (school) |  | IM: -.16  ID: -.30  CG: -.24 | – | – | – | – | IM: < .05  ID: < .01  CG: < .01 | *  **  ** |
|  | regression | PD – well-being (feelings) |  | – | -.11 (.04) | – | – [-0.19–0.02] | – | < .01 | ** |
|  |  | PD – well-being (school) |  | – | -.18 (.04) | – | – [-0.27– -0.10] | – | < .01 | ** |
| ***OTHER MENTAL AND PHYSICAL HEALTH-RELATED OUTCOMES (k=10)*** | | | | | | | | | | |
| Borges (2011) | conditional logistic regression | discrimination (US-born) – deliberate self-injury |  | – | – | – | 3.1 [1.6–5.9] | – | NA |  |
|  |  | discrimination (US-born) – suicidal ideation |  | – | – | – | 2.1 [1.2–3.8] | – | NA |  |
|  |  | discrimination (foreign-born) – deliberate self-injury |  | – | – | – | 1.5 [0.7–3.4] | – | NA |  |
|  |  | discrimination (foreign-born) – suicidal ideation |  | – | – | – | 1.2 [0.6–2.6] | – | NA |  |
| Correa-Velez (2010) | generalized estimating equations | ED – happiness |  |  |  |  | NA | NA | > .05 |  |
| el Bouhaddani (2019) | logistic regression | PD (personal) – overall psychotic experiences |  | – | – | – | 2.30 [1.22–4.34] | – | < .05 | * |
|  |  | PD (personal) – hallucinatory experiences |  | – | – | – | 1.65 [0.73–3.72] | – | > .05 |  |
|  |  | PD (personal) – delusional experiences |  | – | – | – | 2.94 [1.43–6.06] | – | < .01 | ** |
|  |  | PD (group) – overall psychotic experiences |  | – | – | – | 1.56 [0.81–2.99] | – | > .05 |  |
|  |  | PD (group) – hallucinatory experiences |  | – | – | – | 1.53 [0.67–3.52] | – | > .05 |  |
|  |  | PD (group) – delusional experiences |  | – | – | – | 1.32 [0.61–2.85] | – | > .05 |  |
| Ellis (2008) | correlation, multiple linear regression | PD – PTSD |  | .47 | .22 (.07) | .25 | – | – | cor: < .01  reg: < .01 | **  ** |
| Liebkind (2000) | correlation, regression | discrimination – acculturative stress |  | .30 | – | .29 | – | – | cor: < .01  reg: < .001 | **  *** |
| Neto (2000) | correlation | PD – stressful experience adaptation |  | .31 | – | – | – | – | < .01 | ** |
|  |  | PD – psychological symptoms |  | .34 | – | – | – | – | < .01 | ** |
| Pantzer (2006) | multiple regression | PD – health-related quality of life |  | – | -1.19 (.35) | – | – | – | < .001 | *** |
| Potochnick (2010) | logit regression | ED – anxiety |  | – | – | – | 1.22 [0.64–2.30] | – | > .10 |  |
|  |  | PD – anxiety |  | – | – | – | 0.95 [0.61–1.50] | – | > .10 |  |
| Sabatier (2008) | multiple hierarchical regression | PD – stress symptoms (French sample only) |  | – | – | NA | – | – | NA |  |
| Verkuyten (2004) | correlation | PD – global self-worth |  | S1: -.17  S2: -.08 | – | – | – | – | S1: < .05  S2: > .05 | * |
|  | multiple regression | PD – global self-worth |  | – | – | S1: -.16  S2: -.03 | – | – | S1: < .05  S2: > .05 | * |
| **SCHOOL-RELATED OUTCOMES (k=6)** | | | | | | | | | | |
| ***ACADEMIC ACHIEVEMENT AND PERFORMANCE (k=6)*** | | | | | | | | | | |
| Guerra (2019) | correlation | PD – school achievement |  | IM: -.01  ID: -.00  CG: -.10 | – | – | – | – | IM: > .05  ID: > .05  CG: > .05 |  |
|  | regression | PD – school achievement |  | – | -.003 (NA) | – | NA | – | .919 |  |
| Kiang (2015) | hierarchical linear model | PD – perceived academic performance |  | – | NA (.04) | -.05 | – | – | > .05 |  |
| Oczlon (2021) | correlation | PD – academic achievement |  | -.02 | – | – | – | – | > .05 |  |
| Oxman-Martinez  et al. (2012) | correlation, regression | peer discrimination – academic grades |  | -.10 | -.01 (.01) | -.02 | – | – | cor: < .01  reg: > .05 | ** |
|  |  | teacher discrimination – academic grades |  | -.94 | -.02 (.01) | -.05 | – | – | cor: < .01  reg: > .05 | ** |
| Özdemir (2013) | correlation, mediated regression | ethnic harassment (T1) – perceived academic failure |  | T1: .24  T2: .22 | – | NA | – | – | cor (T1, T2):  < .001  reg: NA | *** |
| Verkuyten (2004) | correlation | PD – educational performance |  | S1: -.01  S2: .01 | – | – | – | – | S1: > .05  S2: > .05 |  |
| ***OTHER SCHOOL-RELATED OUTCOMES (k=2)*** | | | | | | | | | | |
| Kiang (2015) | hierarchical linear model | PD – school value (positive) |  | – | NA (.03) | -.11 | – | – | < .01 | ** |
| Oczlon (2021) | correlation | PD – academic self-concept |  | -.15 | – | – | – | – | < .05 | * |
| **OTHER DEVELOPMENTAL OUTCOMES (k=13)** | | | | | | | | | | |
| ***RELATIONSHIPS WITH OTHERS (k=4)*** | | | | | | | | | | |
| Becerra (2015) | ordinal logistic regression | PD – avoiding family activities |  | – | .21 (.12) | – | 1.24 [NA] | – | > .05 |  |
| Kiang (2015) | hierarchical linear model | PD – relationships with others (positive) |  | – | -.20 (.04) | – | – | – | < .001 | *** |
| Neto (2000) | correlation, regression | PD – loneliness |  | .31 | – | .28 | – | *R²* = .26 | cor: < .01  reg: < .01 | **  ** |
| Özdemir (2013) | correlation, mediated regression | ethnic harassment (T1) – positive relationships with teachers (T1) |  | -.26 | – | NA | – | – | < .001 | *** |
| ***MISCONDUCT AND DELINQUENCY (k=4)*** | | | | | | | | | | |
| Cano (2015) | correlation | perceived negative context of reception – rule-breaking behaviour |  | T1: .27  T2: .18 | – | – | – | – | T1: < .01  T2: < .01 | **  ** |
|  |  | bicultural stress – rule-breaking behaviour |  | T1: .56  T2: .36 | – | – | – | – | T1: < .01  T2: < .01 | **  ** |
|  |  | PD – rule-breaking behaviour |  | T1: .35  T2: .32 | – | – | – | – | T1: < .01  T2: < .01 | **  ** |
|  | path analysis | cultural stress^a^ – rule-breaking behaviour |  | – | – | .25 | – | – | < .001 | *** |
| D’hondt (2016) | multilevel regression | teacher ethnic discrimination (non-frequent) – school misconduct |  | – | .10 (.03) | – | – | – | < .001 | *** |
|  |  | teacher ethnic discrimination (frequent) – school misconduct |  | – | .28 (.05) | – | – | – | < .001 | *** |
| Kogan (2019) | regression | racial discrimination – delinquency |  | – | .81 (.13) | .50 | – | – | < .001 | *** |
| Sabatier (2008) | multiple hierarchical regression | PD – deviance |  | – | – | NA | – | *–* | NA |  |
| ***SENSE OF MASTERY AND CONTROL (k=3)*** | | | | | | | | | | |
| Liebkind (2000) | correlation, regression | discrimination – sense of mastery |  | -.11 | – | -.05 | – | – | cor: < .01  reg: > .10 | ** |
| Neto (2000) | correlation | PD – mastery |  | -.18 | – | – | – | – | > .05 |  |
| Verkuyten (1998) | correlation | PD (personal) – sense of control |  | TR: –  MA: -.28 | – | – | – | – | TR: –  MA: < .01 | ** |
|  |  | PD (group) – sense of control |  | TR: –  MA: -.01 | – | – | – | – | TR: –  MA: > .05 |  |
| ***SUBSTANCE USE (k=2)*** | | | | | | | | | | |
| Cano (2015) | correlation | perceived negative context of reception – substance use (T2) |  | cig: .05  alc: .08 | – | – | – | – | cig: > .05  alc: > .05 |  |
|  |  | bicultural stress – substance use |  | cig: .14  alc: .10 | – | – | – | – | cig: < .05  alc: > .05 | * |
|  |  | PD – substance use |  | cig: .18  alc: .25 | – | – | – | – | cig: < .01  alc: < .01 | **  ** |
|  | path analysis | cultural stress^a^ – substance use |  | – | – | – | cig: 1.24 [NA]  alc: 1.20 [NA] | – | cig: < .001  alc: < .001 | ***  *** |
| Okamoto  et al. (2009) | logistic regression | PD – cigarette use |  | – | – | – | 1.73 [1.30–2.31] | – | < .01 | ** |
|  |  | PD – alcohol use |  | – | – | – | 1.53 [1.20–1.96] | – | < .01 | ** |
|  |  | PD – marijuana use |  | – | – | – | 1.70 [1.21–2.40] | – | < .01 | ** |
|  |  | PD – inhalant use |  | – | – | – | 1.50 [1.02–2.25] | – | < .05 | * |
| ***LIFE SATISFACTION (k=2)*** | | | | | | | | | | |
| Liebkind (2000) | correlation, regression | discrimination – life satisfaction |  | -.15 | – | -.09 | – | – | cor: < .01  reg: < .05 | **  * |
| Neto (2001) | correlation, regression | PD – satisfaction with life |  | -.12 | – | -.09 | – | – | cor: < .05  reg: > .05 | * |
| ***SENSE OF COMPETENCE (k=2)*** | | | | | | | | | | |
| Oxman-Martinez  et al.(2012) | correlation, regression | peer discrimination – sense of competence in peer relationships |  | -.22 | -.18 (.05) | -.13 | – | – | cor: < .01  reg: < .001 | **  *** |
|  |  | teacher discrimination – sense of competence in peer relationships |  | -.16 | -.15 (.05) | .10 | – | – | cor: < .01  reg: < .01 | **  ** |
|  |  | peer discrimination – sense of academic competence |  | -.10 | -.02 (.01) | -.05 | – | – | cor: < .01  reg: > .05 | ** |
|  |  | teacher discrimination – sense of academic competence |  | -.10 | -.03 (.02) | -.07 | – | – | cor: < .01  reg: < .05 | **  * |
| Verkuyten (1998) | correlation | PD (personal) – social competence |  | TR: -.23  MA: -.27 | – | – | – | – | TR: < .05  MA: < .05 | *  * |
|  |  | PD (personal) – cognitive competence |  | TR: -.09  MA: -.04 | – | – | – | – | TR: > .05  MA: > .05 |  |
|  |  | PD (personal) – physical competence |  | TR: -.02  MA: -.08 | – | – | – | – | TR: > .05  MA: > .05 |  |
|  |  | PD (group) – social competence |  | TR: -.08  MA: -.13 | – | – | – | – | TR: > .05  MA: > .05 |  |
|  |  | PD (group) – cognitive competence |  | TR: -.01  MA: -.05 | – | – | – | – | TR: > .05  MA: > .05 |  |
|  |  | PD (group) – physical competence |  | TR: -.02  MA: -.15 | – | – | – | – | TR: > .05  MA: > .05 |  |

*Notes*. ^a^ global category of perceived ethnic discrimination, negative context of reception and bicultural stress; alc = alcohol, BC = Black Caribbean, CA = Canada, cig = cigarettes, cor = correlation, cross = cross-sectional effects, ED = experienced discrimination, f = female, FR = France, ID = immigrant descendants IG = index group, IM = immigrants, IN = India, LK = Sri Lanka, long = longitudinal effects, m = male, MA = Morocco, NA = not available, NG/GH = Nigeria & Ghana, oA = other African, PD = perceived discrimination, PK/BD = Pakistan & Bangladesh, PTSD = Posttraumatic Stress Disorder, reg = regression, S1/2 = study 1/2, SEM = structural equation modeling, SO = Somalia, SOA = Strength of Association, T1/2 = timepoint 1/2, TR = Turkey, VN = Vietnam, W1/2 = wave 1/2; reported results were shortened to two decimal places. * p < .05, ** p < .01, *** p < .001.
